# New Prenylxanthones from the Deep-Sea Derived Fungus *Emericella* sp. SCSIO 05240

**DOI:** 10.3390/md12063190

**Published:** 2014-05-28

**Authors:** Mangaladoss Fredimoses, Xuefeng Zhou, Xiuping Lin, Xinpeng Tian, Wen Ai, Junfeng Wang, Shengrong Liao, Juan Liu, Bin Yang, Xianwen Yang, Yonghong Liu

**Affiliations:** Key Laboratory of Tropical Marine Bio-resources and Ecology, Guangdong Key Laboratory of Marine Materia Medica, RNAM Center for Marine Microbiology, South China Sea Institute of Oceanology, Chinese Academy of Sciences, Guangzhou 510301, China; E-Mails: moses10c@gmail.com (M.F.); xfzhou@scsio.ac.cn (X.Z.); xiupinglin@hotmail.com (X.L.); xinpengtian@scsio.ac.cn (X.T.); aiwen747@gmail.com (W.A.); Junfeng1982a@163.com (J.W.); ljrss@126.com (S.L.); ljuan2010@qq.com (J.L.); bingo525@163.com (B.Y.); xwyang@scsio.ac.cn (X.Y.)

**Keywords:** prenylxanthone, *Emericella*, deep-sea, fungus

## Abstract

Four new prenylxanthones, emerixanthones A–D (**1**–**4**), together with six known analogues (**5**–**10**), were isolated from the culture of the deep-sea sediment derived fungus *Emericella* sp. SCSIO 05240, which was identified on the basis of morphology and ITS sequence analysis. The newstructures were determined by NMR (^1^H, ^13^C NMR, HSQC, HMBC, and ^1^H-^1^H COSY), MS, CD, and optical rotation analysis. The absolute configuration of prenylxanthone skeleton was also confirmed by the X-ray crystallographic analysis. Compounds **1** and **3** showed weak antibacterial activities, and **4** displayed mild antifungal activities against agricultural pathogens.

## 1. Introduction

Naturally occurringxanthones, dibenzo-γ-pyrone derivatives produced by higher plants, lichens, and fungi, contain different substituents on the two benzene rings, thus resulting in large structural diversity [1,2]. These substituents are strongly dependent on their biosynthetic origins and modification reactions [3]. Prenylxanthones, an important subgroup of naturally occurring xanthones, exhibit diverse biological and pharmaceutical activities, because of their specific substitution patterns [4].In many conditions, activities are associated with prenylation of the xanthone skeleton [5].Various biological and pharmacological activities, such as antibacterial, antifungal, anti-inflammatory, antioxidant, and antitumor, have been reported for prenylxanthones, which make these compounds attractive for pharmaceutical applications [6].

Marine fungi are known to be a prolific source of biologically active natural products which might be useful for drug discovery [7]. As a special ecosystem, marine sediment provides an abundant of fungal resources, which yielded various secondary metabolites with novel structures and interesting biological activities [8]. The genus *Emericella* fungi could produce a remarkable diversity of secondary metabolites, such as indole alkaloids, prenylated polyketides, benzophenones, and xanthones, with interesting biological properties thus representing potential leads for the developing of new pharmaceutical agents [9,10]. As part of our research program to discover new natural products from marine deep-sea sediment fungi from South China Sea, we have isolated four new (**1**–**4**) and six known (**5**–**10**) prenylxanthones, from the culture of a deep-sea sediment derived fungus *Emericella* sp. SCSIO 05240.

## 2. Results and Discussion

### 2.1. Identification of the Fungus Strain

**Figure 1:**
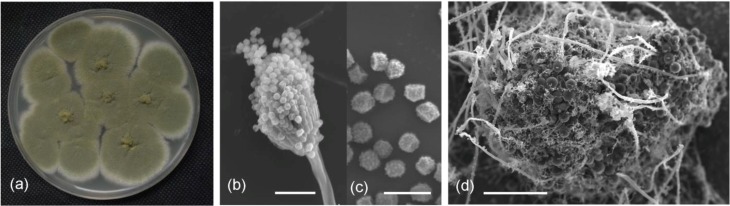
Colony appearance and micromorphology of strain SCSIO 05240. (**a**) Colony appearance after 7 days at 25 °C (PDA); (**b**) Conidiophores under SEM; (**c**) Conidia under SEM; (**d**) Cleistothecia as seening using SEM. Bars: 15 μM (**b**), 5 μM (**c**), and 50 μM (**d**).

A fungal isolate from deep-sea sediment (3258 m) of the South China Sea displayed activity against bacteria and fungi in our previous screening tests. The cultural and morphological properties suggested that the isolate, termed SCSIO 05240, was a strain of *Emericella* (Figure 1a–d). Abundant conidiophores and cleistothecia occurred on PDA medium. Conidial heads were mostly short cylindrical. Vesicles were hemispherical and biseriate. Metulae were smoothing walled and cylindrical. Phialides were smooth walled and flask shaped, approximately equal in length to those of metulae. Conidia were olive green and globose to subglobose when mature, and spiny (Figure 1b,c). Cleistothecia were spherical after mature, and were typically surrounded by thickened large spherical envelop cells (Figure 1d).

**Figure 2:**
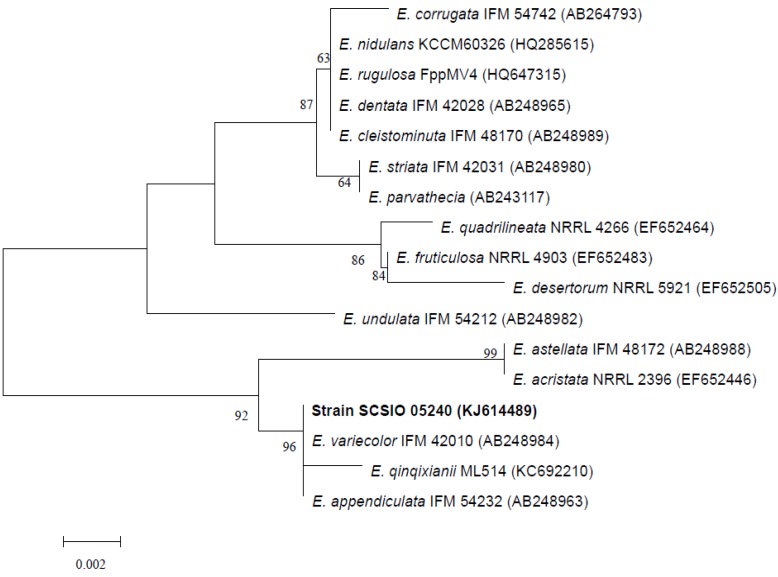
Neighbor-joining tree based on sequences of ITS region, showing phylogenetic relationships between *Emericella* sp. SCSIO 05240 and related *Emericella* species. Numbers at nodes indicate bootstrap values from 1000 replicates. GenBank accession numbers are given in *parentheses*. Bar, 0.2% sequence divergence.

ITS1-5.8S-ITS2 sequence region (507 basepairs (bp), accession number KJ614489) of strain SCSIO 05240 was obtained and was found to be most similar to those of *E.qinqixianii* ML514, *E. variecolor* IFM 42010, and *E. appendiculata* IFM 54232, with sequence identity of 99%. A phylogenetic tree was constructed, using the neighbor-joining method based on similarity of a 506-bp consensus length of ITS1-5.8S-ITS2 sequence (Figure 2), and confirmed that strain SCSIO 05240 grouped most closely with *E. variecolor* IFM 42010. The ITS region sequence identity confirmed that strain SCSIO 05240 belonged to genus *Emericella*, and was designated as *Emericella* sp. SCSIO 05240.

### 2.2. Structure Elucidation

**Figure 3:**
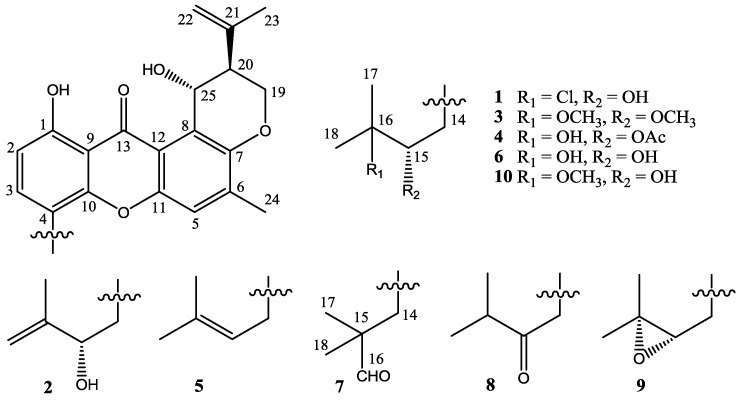
Chemical structures of compounds **1**–**10**.

**Figure 4:**
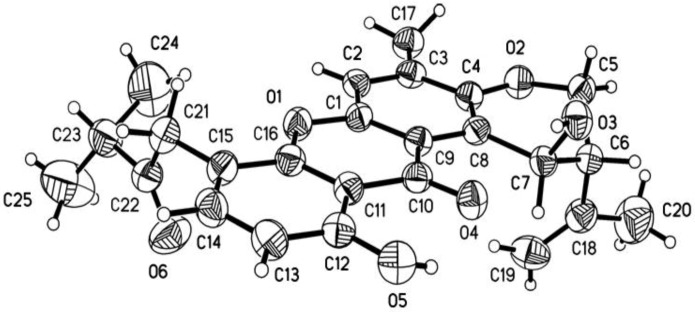
X-ray structure of compound **8**.

Four new prenylxanthones, emerixanthones A–D (**1**–**4**), together with six known analogues (**5**–**10**), were isolated and purified from the cultures of SCSIO 05240. Six known prenylxanthones were identified as shamixanthone (**5**) [11], tajixanthone hydrate (**6**) [11], ruguloxanthone A (**7**) [12], ruguloxanthone B (**8**) [12], tajixanthone (**9**) [13], and tajixanthone methonate (**10**) [11], by comparison with their ^1^H and ^13^C NMR with those reported (Figure 3). In order to confirm the absolute configuration of prenylxanthones skeleton, the X-ray crystallographic analysis of ruguloxanthone B (**8**) was carried out (Figure 4). It is the first X-ray crystallographic analysis of the prenylxanthones derivatives.

**Figure 5:**
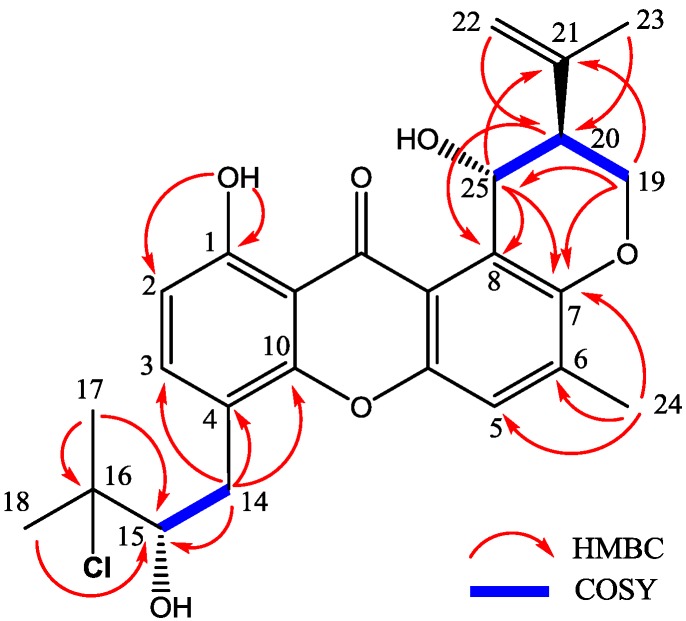
Key HMBC and COSY correlations of compound **1**.

Compound **1** was obtained as yellow needle crystals and was assigned the molecular formula C_25_H_27_ClO_6_, as deduced from HRESIMS data (*m*/*z* 459.1569 [M + H]^+^, calcd for 459.1574), indicating 12 degrees of unsaturations. The IR spectrum showed the presence of hydroxyl 3394 (OH), 2978, 2916, and aromatic ketone 1639 and 1573 (C=O), 1473, 1431, 1354, 1292, 1242, 1049 and 1018 (C–O–C), 856, and 821 cm^−1^. The ^1^H and ^13^C NMR spectra (Table 1 and Table 2) and DEPT experiments suggested that the presence of 25 carbon signals, which were attributable to four methyls, three methylenes, six methines (including aromatic and chlorinated carbons), and nonprotonated (including one ketone carbonyl) carbons. The ^1^H NMR spectroscopic data has showed resonances for three aromatic protons, with two (δ_H_ 6.78, H-2 and δ_H_ 7.55, H-3) exhibiting *ortho* coupling (*J* = 8.5 Hz) and the third (δ_H_ 7.22, H-5) appearing as a singlet. The aromatic methyl substituent (δ_H_ 2.35, s, H-24) was connected to position C-6, as supported by HMBC correlations of CH_3_-24 to C-5, C-6 and C-7. A chelated hydroxyl resonance at δ_H_ 12.63 was assigned to the aromatic carbon atom C-1, as confirmed by HMBC correlations from OH-1 to C-1 and C-2. The OCHCHCH_2_O spin system, corresponding to the C-19/C-20/C-25 unit of **1**, was consistent with a dihydropyran ring fused to an aromatic ring at the position of C-7 and C-8, which was also confirmed by the HMBC and ^1^H-^1^H COSY correlations showed in Figure 5. The two terminal olefinic proton signals, together with a methyl singlet at δ_H_ 1.84 (3H, s, H-23), revealed that an isopropenyl moiety was connecting to the pyran ring at C-20 and was confirmed by HMBC correlation of H_3_-23 and H_2_-22 to C-20. These data indicated the same prenylxanthones skeleton as the known derivative ruguloxanthone B (**8**), whose absolute configuration has been confirmed by X-ray.

**Table 1:** ^1^H NMR spectroscopic data of compounds **1**–**4** (δ_H_, mult, *J*_H_, 500 MHz, CDCl_3_).

No.	1	2	3	4
2	6.78, d, 8.5	6.76, d, 8.5	6.78, d, 8.5	6.71, d, 8.5
3	7.55, d, 8.5	7.51, d, 8.5	7.59, d, 8.5	7.41, d, 8.5
5	7.22, s	7.26, s	7.22, s	7.25, s
14a	3.35, brd, 14.0	3.18, dd, 14.0, 5.0	3.14, brd, 14.0	3.34, dd, 14.0, 2.5
14b	2.76, dd, 14.0, 10.5	2.97, dd, 14.0, 8.5	2.72, dd, 14.0,10.0	2.91, dd,14.0, 10.5
15	3.84, brd, 10.5	4.39, m	3.78, d, 10.0	5.17, dd, 10.5, 7.5
17	1.76, s	4.84, s,4.91,s	1.32, s	1.37, s
18	1.73, s	1.88, s	1.28, s	1.33, s
19a	4.43, dd, 10.5, 2.5	4.43, dd, 11.0, 2.5	4.43, dd, 11.0, 3.0	4.43, dd, 10.5, 3.0
19b	4.35, dd, 10.5,2.5	4.35, dd, 11.0, 2.5	4.36, dd, 11.0, 3.0	4.35, dd, 10.5, 3.0
20	2.73, brs	2.73, brd, 3.0	2.73, brd, 2.5	2.73, brd, 2.5
22a	4.79, s	4.80, s	4.80, s	4.79, s
22b	4.56, s	4.58, s	4.57, s	4.56, s
23	1.84, s	1.85, s	1.84, s	1.86, s
24	2.35, s	2.35, s	2.35, s	2.35, s
25	5.40, brs	5.41, brd 2.5	5.41, brs	5.40, brd, 2.5
15-OCH_3_			3.48, s	
16-OCH_3_			3.29, s	
OAc				1.84, s
OH-1	12.63, s	12.65, s	12.62, s	12.63, s
OH-25	5.0, brs	5.03, d, 4.0	5.05, d, 3.5	4.97, d, 3.5

**Table 2:** ^13^C NMR spectroscopic data of compounds **1**–**4** (δ_C_, mult, 125 MHz, CDCl_3_).

No.	1	2	3	4	No.	1	2	3	4
1	160.5 s	160.4 s	160.2 s	160.6 s	16	74.9 s	146.8 s	76.5 d	72.3 s
2	110.0 d	109.9 d	109.9 d	109.6 d	17	28.9 q	111.4 t	22.5 q	26.9 q
3	138.3 d	138.2 d	138.2 d	137.8 d	18	27.9 q	18.0 q	20.9 q	25.2 q
4	115.9 s	115.6 s	116.8 s	115.1 s	19	64.6 t	64.6 t	64.6 t	64.5 t
5	119.1 d	119.1 d	119.1 d	119.2 d	20	44.9 d	44.9 d	44.9 d	44.9 d
6	138.5 s	138.5 s	138.3 s	138.5 s	21	142.5 s	142.5 s	142.6 s	142.6 s
7	149.6 s	149.5 s	149.5 s	149.6 s	22	112.3 t	112.3 t	112.3 t	112.2 t
8	121.1 s	121.1 s	121.1 s	121.9 s	23	22.5 q	22.5 q	19.3 q	20.7 q
9	109.2 s	109.2 s	109.2 s	109.2 s	24	17.4 q	17.4 q	17.4 q	17.4 q
10	153.1 s	153.2 s	153.0 s	153.2 s	25	63.2 d	63.2 d	63.2 d	63.1 d
11	152.0 s	152.0 s	152.1 s	152.0 s	15-OCH_3_			29.7 q	
12	116.9 s	116.8 s	116.9 s	116.9 s	16-OCH_3_			49.3 q	
13	184.4 s	184.4 s	184.5 s	184.4 s	OAc(CO)				170.3 s
14	31.9 t	35.3 t	31.2 t	29.6 t	OAc(CH_3_)				20.7 q
15	78.5 d	75.4 d	77.6 s	78.6 d					

The isotopic ion peaks signals (3:1) in the ESIMS spectrum of **1** indicating the presence of a chlorine atom in the molecule. The isopentyl moiety connected to position C-4, which is different from ruguloxanthone B (**8**) and taxjixanthone hydrate (**6**), was deduced as 3-chloro-3-methyl-2-hydroxy butyl moiety, by the HMBC and ^1^H-^1^H COSY correlations showed in Figure 5. The chemical shifts of H-14a, H-14b/C-14 and H-15/C-15 (δ_H_/δ_C_ 3.35, 2.76/31.9 and 3.84/78.5) also suggest C-15-OH, C-16-Cl replacement of **1**, rather than C-15-Cl, C-16-OH moiety [9].

The configurations at C-20, C-25 of **1** are the same as those of ruguloxanthone B (**8**), whose absolute configuration has been confirmed by X-ray. The configuration at C-15 is same as taxjixanthone hydrate (**6**), on the basis of comparison of optical rotation data [−78.1 (*c* 0.0031, CHCl_3_)] of **1** and [−76.0 (*c* 0.23, CHCl_3_)] of **6** [11]. Thus, the compound was determined as 16-chlorotajixanthone hydrate, as shown in Figure 5, and named as emerixanthone A (**1**).

**Figure 6:**
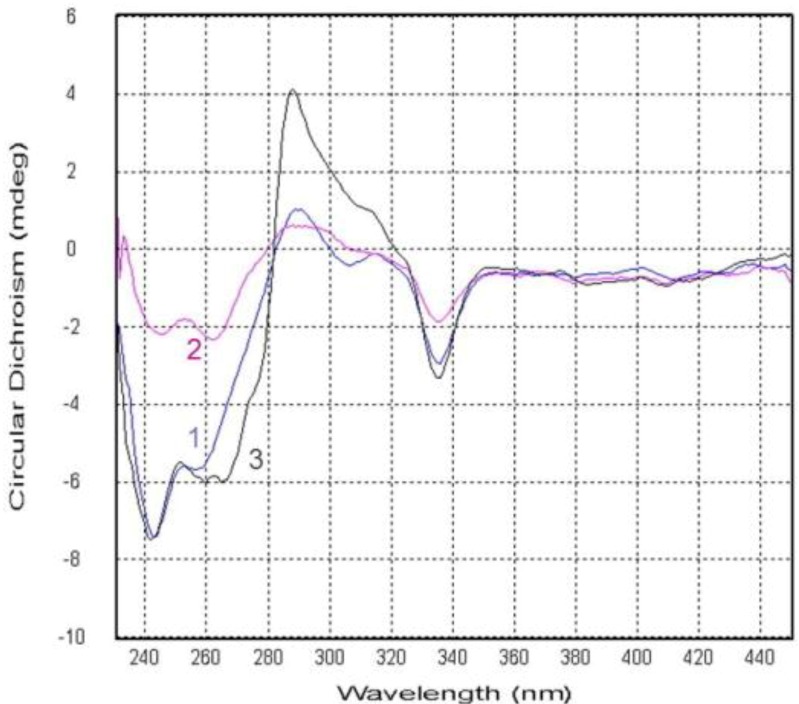
Circular dichroism spectra of compounds **1**–**3**.

Compound **2** was obtained as yellow crystals and was assigned the molecular formula C_25_H_26_O_6_, as deduced from HRESIMS data (*m*/*z* 423.1804 [M + H]^+^, calcd for 423.1808), indicating 13 degrees of unsaturations. The IR spectrum showed the presence of hydroxyl 3444 (OH), 2954, and conjugated ketone groups 1724, 1639, 1604, 1573 (C=O), 1469, 1413, 1350, 1273, 1195, 1049, and 1018 (C–O–C) 902, and 864 cm^−1^. The ^1^H and ^13^C NMR spectra of **2** (Table 1 and Table 2) were similar to those of **1** except for the absence of a 3-chloro-3-methyl-2-hydroxy butyl side chain, which was replaced by a 3-methyl-2-hydroxy-but-3-enyl side chain. The HMBC spectrum showed the correlations of H_2_-14 to C-3, C-4, C-10, C-15, and C-16; H-15 to C-16 and C-18; H_3_-17 to C-15 and C-18, which supported the presence of this side chain. Compound **2** showed a negative specific optical rotation [−54.3 (*c* 0.0065, CHCl_3_)], in contrast to the related compound ruguloxanthone C [+71 (*c* 0.06 CHCl_3_)], which were reported to as an *R* configuration at C-15 [12]. On the basis of this evidence as well as the comparison of CD spectrum of **1** with **2** (Figure 6), 15*S*, 20*S*, 25*R* configuration of **2** was confirmed, as same as that of **1**. The new prenylxanthone was named as emerixanthone B (**2**).

Compound **3** was obtained as yellow needle crystals and was assigned the molecular formula C_27_H_32_O_7_, as deduced from HRESIMS data (*m*/*z* 469.2217 [M + H]^+^, calcd for 469.2226), indicating 12 degrees of unsaturations. The IR spectrum showed the presence of hydroxy 3429 (OH), 2920, and aromatic ketone 1639 and 1573 (C=O), 1473, 1431, 1292, 1149, 1049, 1018 (C–O–C), and 856 cm^−1^. The NMR spectra revealed the same prenylxanthone skeleton of **3** as those of **1**, **2** and **8**. The ^1^H and ^13^C NMR spectra of **3** (Table 1 and Table 2) were much similar to those of tajixanthone methonate (**10**) [11] except for the additional methoxy signal δ_H_ 3.48 (3H, s, OCH_3_) corresponding to C-15. One more methoxy signal δ_H_ 3.29 (3H, s, OCH_3_) was found to be connected to C-16, as same as that of **10**. Thus, the side chain of **3** was deduced as 2, 3-dimethoxy-3-methyl butyl, which is confirmed by the HMBC spectrum. Compound **3** also exhibited negative specific optical rotation [−95.5 (*c* 0.0018, CHCl_3_)] in the same manner as **1**. The comparison of CD spectrum of **3** with **1** and **2** (Figure 6) also showed the same configuration of **3**. This is the third new prenylxanthone,15,16-dimethoxytajixanthone hydrate, named as emerixanthone C (**3**).

Compound **4** was obtained as yellow needle crystals and was assigned the molecular formula C_27_H_30_O_8_, as deduced from HRESIMS data (*m*/*z* 483.2015 [M + H]^+^, calcd for 483.2019), indicating 13 degrees of unsaturations. The NMR spectra revealed the same prenylxanthone skeleton of **4** as those of **1**–**3**. The ^1^H and ^13^C NMR spectra of **4** (Table 1 and Table 2) were much close to those of tajixanthone hydrate (**6**) [11], except for the additional acetate group (δ_C_ 170.3, 20.7; δ_H_ 1.84, 3H, s) connected to C-15 (δ_C_ 78.6; δ_H_ 5.17, 1H, dd, *J* = 10.5, 3.0 Hz). The multiplicity and coupling constant values for H-14a, H-14b, and H-15 of **4** were very similar to those of **3** and **6**, suggesting the same relative stereochemistry for these compounds. The optical rotation data [−93.3 (*c* 0.009, CHCl_3_)] also confirmed the same configuration of **4** as **3**. Compound **4** was deduced as taxjixanthone hydrate-15-acetate (**4**). Tajixanthone hydrate (**6**) was stable and no change has been found subject to the extraction conditions, and taxjixanthone hydrate-15-formate was also reported as natural product [14]. Accordingly we believe that **4** is a true natural product, named as emerixanthone D (**4**).

### 2.3. Biological Activities of the Isolated Compounds

The isolated prenylxanthone derivatives **1**–**10** were evaluated for antibacterial, antifungal, and antitumor activities. Compounds **1** and **3** showed weak antibacterial activity against all pathogens *Escherichia coli* (ATCC 29922), *Klebsiella pneumonia* (ATCC 13883), *Staphylococcus aureus* (ATCC 29213), *Enterococcus faecalis* (ATCC 29212), *Acineto* *bacterbaumannii* (ATCC 19606), and *Aeromonas* *hydrophila* (ATCC 7966). The diameters of inhibition zones of **1** and **3** against sixpathogens were all 4–6 mm, while that of the positive control ciprofloxacin was about 35–40 mm. Compound **4** also showed mild antifungal activity against all agricultural pathogens *Fusarium* sp., *Penicillium* sp., *Aspergillus* *niger*, *Rhizoctonia* *solani*, *Fusariumoxy* *sporium f.* sp. *niveum*, *Fusariumoxy* *sporium f.* sp. *cucumeris*, diameters of inhibition zones of which were both 3–4 mm. The diameters of inhibition zones of the positive control, carbendazim, were about 40–45 mm. None of them displayed inhibitory activity against ten human tumor cell lines (K562, A549, HL60, Huh-7, MCF-7, H1975, U937, BGC-823, Hela, and Molt-4).

### 2.4. Discussion

**Figure 7:**
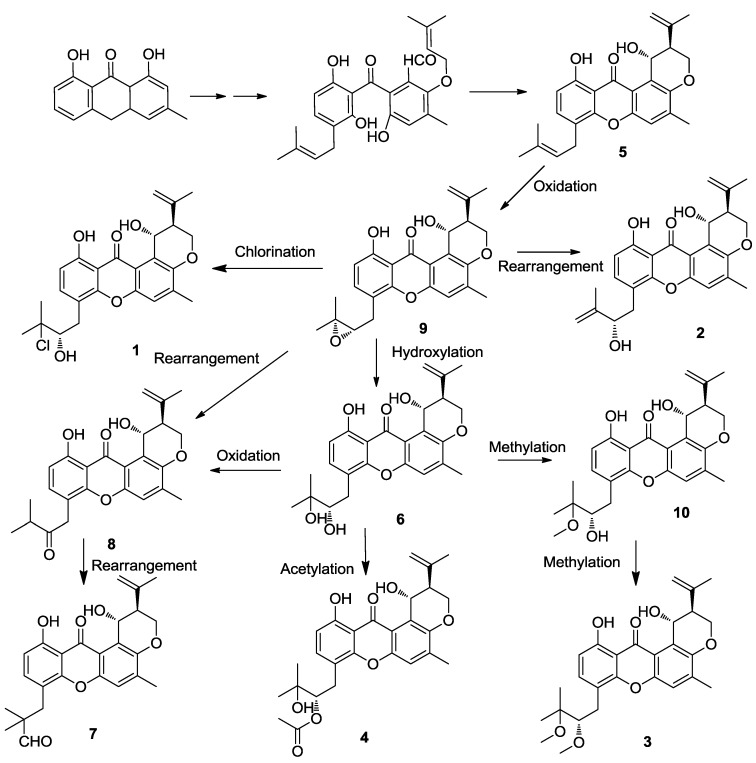
Proposed biosynthetic pathway of compounds **1**–**10**.

Prenylxanthone derivatives have also been found in other *Emericella* fungi, such as *E.* *rugulosa* [12] and *E.* *variecolor* (*Aspergillus* *variecolor*) [11,13]. The biosynthesis of tajixanthone (**9**) and related metabolites of *A.* *variecolor* has been studied by incorporation experiments, and it provided further circumstantial evidence that this group of metabolites was biogenetically related to tajixanthone (**9**) and shamixanthone (**5**) [15]. The formation of shamixanthone (**5**) occurs by cyclodehydration of the dihydroxybenzophenone system present as a two hemiketal forms as arugosis A and B, could be an intermediate in the biosynthesis of tajixanthone, being formed through oxidative ring cleavage of a suitably substituted anthraquinone. Subsequent cyclodehydration and intramolecular ‘ene’ cyclization of the orthoprenyloxy aldehyde moiety would provide the xanthone nucleus and the substituted dihydropyran ring respectively. From this result, compounds **1**–**4** were obtained through the tajixanthone biosynthetic pathway under various modification of such as hydroxylation, oxidation, chlorination, acetylation, methylation, and rearrangement (Figure 7).

## 3. Experimental Section

### 3.1. General

Optical rotations were measured with a perkine Elmer 341 polarimeter. Circular dichroism spectra were measured with a Chirascan circular dichroism spectrometer (Applied Photophysics, Ltd., Leatherhead, UK). NMR spectra were obtained on a Bruker AVANCE-500 spectrometer (Bruker, Karlsruhe, Germany) with TMS as internal standard, and chemical shifts were recorded as ä-values. HRESIMS were performed on a Q-Tof Micro mass spectrometer (Waters, Manchester, UK). Silica gel (100–200 and 200–300 mesh), and Sephadex LH-20 for column chromatography were purchased from Qingdao Marine Chemical Group Co. (Qingdao, China), and GE Healthcare (Uppsala, Sweden), respectively.All solvents used were of analytical grade (Tianjin Fuyu Chemical and Industry Factory, Tianjin, China).

### 3.2. Fungal Material and Fermentation

The strain SCSIO 05240 was isolated from a sediment sample (E 120°0.975′, N 19°0.664′) at the depth of 3258 m collected from an open voyage to the South China Sea in August 2007, and was deposited in the type culture collection of Center for Marine Microbiology, Research Network of Applied Microbiology, South China Sea Institute of Oceanology, CAS, Guangzhou, China. The fungus was identified by sequence analysis of the ITS1-5.8S-ITS2 sequence region and micromorphology as described previously [16].

The pure strain SCSIO 05240 colonies were sub-cultured in 100 mL Erlenmeyer flasks each containing 25 mL of malt media (20 g/L of malt extract, 1 g/L of peptone, and 30 g/L of sea salt). For a large scale culture, 25 mL of the seed culture was transferred to a 1000 mL Erlenmeyer flask containing solid rice medium (200 g rice, 200 mL of water, 3% sea salt), and incubated at 28°C under static stations and daylight. After one month, cultures from 30 flasks were harvested for isolation of compounds with antimicrobial activity.

### 3.3. Extraction and Isolation

The culture medium containing the mycelium was cut into small pieces and extracted three times with EtOAc (3 × 6 L). The combined EtOAc extract was filtered and evaporated to afford 120 g of crude extract. The crude EtOAc extract was partitioned between hexanes and 90% MeOH. The resulting MeOH fraction (42.5 g) was subjected to silica gel column chromatography (CC) eluted with petroleum ether/EtOAc in linear gradient (90:10, 80:20, 50:50, 25:75) and followed by CHCl_3_/MeOH in linear gradient (90:10, 80:20, 70:30, 60:40, 0:100) to obtained 18 fractions (fr.1–18) on the basis of TLC profiles. Fr.8 (280 mg) was subjected to Sephadex LH-20 (CHCl_3_/MeOH 1:1) to furnish four sub-fractions, designated as fr.8-1 to 8-4. Fr.8-2 (165.2 mg) was further purified by CC on silica gel (CHCl_3_/MeOH, 40:1) to give **1** (120.4 mg) and **5** (16.7 mg). Fr.6 (240 mg) was purified by Sephadex LH-20 (CHCl_3_/MeOH, 1:1) to give four sub-fractions, labeled as fr.6-1 to 6-4). Fr.6-3 (127 mg) was further purified by CC on silica gel (CHCl_3_/MeOH, gradient elution 100:1~10:1) to afford **2** (13.1 mg) and **6** (38.6 mg). Fr.7 (210 mg) was separated by Sephadex LH-20 (CHCl_3_/MeOH 1:1) to obtained three fractions termed as fr.7-1 to 7-3. Fr.7-2 (84.2 mg) was further purified by HPLC to give **8** (13.7 mg) and **9** (17.4 mg). Fr.12 (276 mg) was separated by Sephadex LH-20 (CHCl_3_/MeOH 1:1) to furnish four sub-fractions named as fr.12-1 to 12-4. Fr.12-1 (22.8 mg) was further purified by HPLC to give **3** (7.5 mg). Fr.12-2 (186 mg) was further purified by CC on silica gel (CHCl_3_/MeOH, gradient elution 100:1~10:1) to give **10** (108.2 mg) and **7** (12.3 mg). Fr.14 (1 g) was subjected to Sephadex LH-20 (CHCl_3_/MeOH 1:1) to obtained four sub-fractions, designated as fr.14-1 to 14-4. Compound **4** (24.4 mg) was obtained by the separation of fr.14-3 on a reversed-phase HPLC eluting with 75% MeOH.

Emerixanthone A (**1**): Yellow needle crystals; 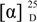 −78.1 (*c* 0.0031, CHCl_3_); UV(CHCl_3_) λ_max_ (log ε) 395 (3.39), 298 (3.63), 294 (3.62), 277 (4.21), 248 (4.04), 228 (4.33) nm; IR ν_max_ 3394, 2978, 2916, 1639, 1573, 1473, 1431, 1354, 1292, 1242, 1049 , 1018, 856 , 821 cm^−1^; ^1^H and ^13^C NMR data see Table 1 and Table 2; HRESIMS *m*/*z* 459.1569 [M + H]^+^ (calcd for C_25_H_28_ClO_6_, 459.1574) (Supplementary Figures S1–S9).

Emerixanthone B (**2**): Yellow crystals; 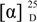 −54.3 (*c* 0.0065, CHCl_3_); UV(CHCl_3_) λ_max_ (log ε) 397 (3.45), 300 (3.63), 292 (3.59), 277 (4.14), 242 (3.88), 228 (3.67) nm; IR ν_max_ 3444, 2954, 1724, 1639, 1604, 1573, 1469, 1413, 1350, 1273, 1195, 1049, 1018, 902, 864 cm^−1^; ^1^H and ^13^C NMR data see Table 1 and Table 2; HRESIMS *m*/*z* 423.1804 [M + H]^+^ (calcd for C_25_H_27_O_6_, 423.1808) (Supplementary Figures S10–S15).

Emerixanthone C (**3**): Yellow needle crystals; 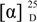 −95.5 (*c* 0.0018 CHCl_3_); UV(CHCl_3_) λ_max_ (log ε) 398 (3.21), 300 (3.41), 291 (3.34), 277 (4.0), 249 (3.16), 228 (4.21) nm; IR ν_max_ 3429, 2920, 1639, 1573, 1473, 1431, 1292, 1149, 1049, 1018, 856 cm^−1^; ^1^H and ^13^C NMR data see Table 1 and Table 2; HRESIMS *m*/*z* 469.2217 [M + H]^+^ (calcd for C_27_H_33_O_7_, 469.2226) (Supplementary Figures S16–S20).

Emerixanthone D (**4**): Yellow needle crystals; 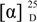 −93.3 (*c* 0.009 CHCl_3_); UV(CHCl_3_) λ_max_ (log ε) 396 (3,61), 299 (3.79), 291 (3.74), 277 (4.32), 249 (3.84), 242 (3.96), 228 (3.29) nm; IR ν_max_ 3479, 2924, 1708, 1643, 1573, 1469, 1242, 1114, 1033, 987, 856 cm^−1^; ^1^H and ^13^C NMR data see Table 1 and Table 2; HRESIMS *m*/*z* 483.2015 [M + H]^+^ (calcd for C_27_H_31_O_8_, 483.2014) (Supplementary Figures S21–S28).

### 3.4. X-ray Crystallographic Analysis of Ruguloxanthone B (8)

Yellow crystal of C_25_H_26_O_6_, *M* = 422.46. Unit cell dimensions *a* = 12.7104(9) Å, *b* = 28.731(3) Å, *c* = 6.0033(3) Å, *V* = 2192.3(3) Å^3^, *Z* = 4; crystal size 0.40 × 0.09 × 0.05 mm^3^. A total of 3769 unique reflections (*θ* = 66.02°–99.00°) were collected using graphite monochromated Cu *K*α radiation (*λ* = 0.71073 Å) on a Bruker Smart 1000 CCD diffractometer at 150(2) K. Absorption corrections were done by semi-empirical from equivalents. The structure was solved by direct methods (SHELXS-97) and refined with Full-matrix least-squares on 3769 data, 0 restraints and 287 variable parameters. Final *R* indicates *R*_1_ = 0.0946, *wR*_2_ = 0.1414[*I* > 2*σ* (I)]. Crystallographic data for **8** has been deposited in the Cambridge Crystallographic Data Centre with the deposition number 992736. Copies of the data can be obtained, free of charge, on application to the director, CCDC, 12 Union Road, Cambridge CB21EZ, UK (Fax: +44-(0)1223-336033, or E-Mail: deposit@ccdc.cam.ac.uk).

### 3.5. Cytotoxic, Antibacterial, and Antifungal Assay

The cytotoxic activities against ten human tumor cell lines, K562, A549, HL60, Huh-7, MCF-7, H1975, U937, BGC-823, Hela, and Molt-4, were determined according to previously reported methods [17]. Antibacterial and antifungal assay against *Escherichia coli* (ATCC 29922*)*, *Klebsiella pneumonia* (ATCC 13883), *Staphylococcus aureus* (ATCC 29213), *Enterococcus faecalis* (ATCC 29212), *Acinetobacter* *baumannii* (ATCC 19606), *Aeromonas* *hydrophila* (ATCC 7966) and pathogens *Fusarium* sp., *Penicillium* sp., *Aspergillus* *niger*, *Rhizoctonia* *solani*, *Fusariumoxy* *sporium f.* sp. *niveum*, *Fusariumoxy* *sporium f.* sp. *Cucumeris* were carried out using the filter paper discagar diffusion method [18]. 25 Microliters (50 μg/mL) of the compound were impregnated on sterile filter paper discs (6 mm diameter). Ciprofloxacin and carbendazim were used as a positive control.

## 4. Conclusions

In summary, four new prenylxanthones (**1**–**4**), including a chlorine-containing prenylxanthone, and six known analogues (**5**–**10**), were isolated from the deep-sea sediment derived fungus *Emericella* sp. SCSIO 05240. Their structures were determined on the interpretation of the NMR, MS, X-ray, OR and CD analysis. Compounds **1**, **3** and **4** exhibited weak antibacterial or antifungal activities.

## Supplementary Files

Supplementary File 1 Supplementary Information (PDF, 2241 KB)
