# Supplementary material for: New Prenylxanthones from the Deep-Sea Derived Fungus Emericella sp. SCSIO 05240
Source: Mar Drugs. 2014 May 28;12(6):3190–202. doi: 10.3390/md12063190 (PMC4071571; doi:10.3390/md12063190)
Supplement: Supplementary File 1 — Supplementary Information (PDF, 2241 KB) [file marinedrugs-12-03190-s001.pdf]

Supplementary Information

Figure S1. ^{1}H NMR (500 MHz, $CDCl_3$) spectrum of compound **1**.
Figure S2. ^{13}C NMR (125 MHz, $CDCl_3$) spectrum of compound **1**.
Figure S3. Dept 135 (125 MHz, $CDCl_3$) spectrum of compound **1**.
Figure S4. HSQC spectrum of compound **1**.
Figure S5. HMBC spectrum of compound **1**.
Figure S6. ^{1}H-^{1}H COSY spectrum of compound **1**.
Figure S7. UV spectrum of compound **1**.
Figure S8. IR spectrum of compound **1**.
Figure S9. HRESI-MS of compound **1**.
Figure S10. ^{1}H NMR (500 MHz, $CDCl_3$) spectrum of compound **2**.
Figure S11. ^{13}C NMR (125 MHz, $CDCl_3$) spectrum of compound **2**.
Figure S12. Dept 135 (125 MHz, $CDCl_3$) spectrum of compound **2**.
Figure S13. UV spectrum of compound **2**.
Figure S14. IR spectrum of compound **2**.
Figure S15. HRESI-MS of compound **2**.
Figure S16. ^{1}H NMR (500 MHz, $CDCl_3$) spectrum of compound **3**.
Figure S17. ^{13}C NMR (125 MHz, $CDCl_3$) spectrum of compound **3**.
Figure S18. UV spectrum of compound **3**.
Figure S19. IR spectrum of compound **3**.
Figure S20. HRESI-MS of compound **3**.
Figure S21. ^{1}H NMR (500 MHz, $CDCl_3$) spectrum of compound **4**.
Figure S22. ^{13}C NMR (125 MHz, $CDCl_3$) spectrum of compound **4**.
Figure S23. Dept 135 (125 MHz, $CDCl_3$) spectrum of compound **4**.
Figure S24. HSQC spectrum of compound **4**.
Figure S25. HMBC spectrum of compound **4**.
Figure S26. UV spectrum of compound **4**.
Figure S27. IR spectrum of compound **4**.
Figure S28. HRESI-MS of compound **4**.

Figure S1. ^{1}H NMR (500 MHz, $CDCl_3$) spectrum of compound **1**.

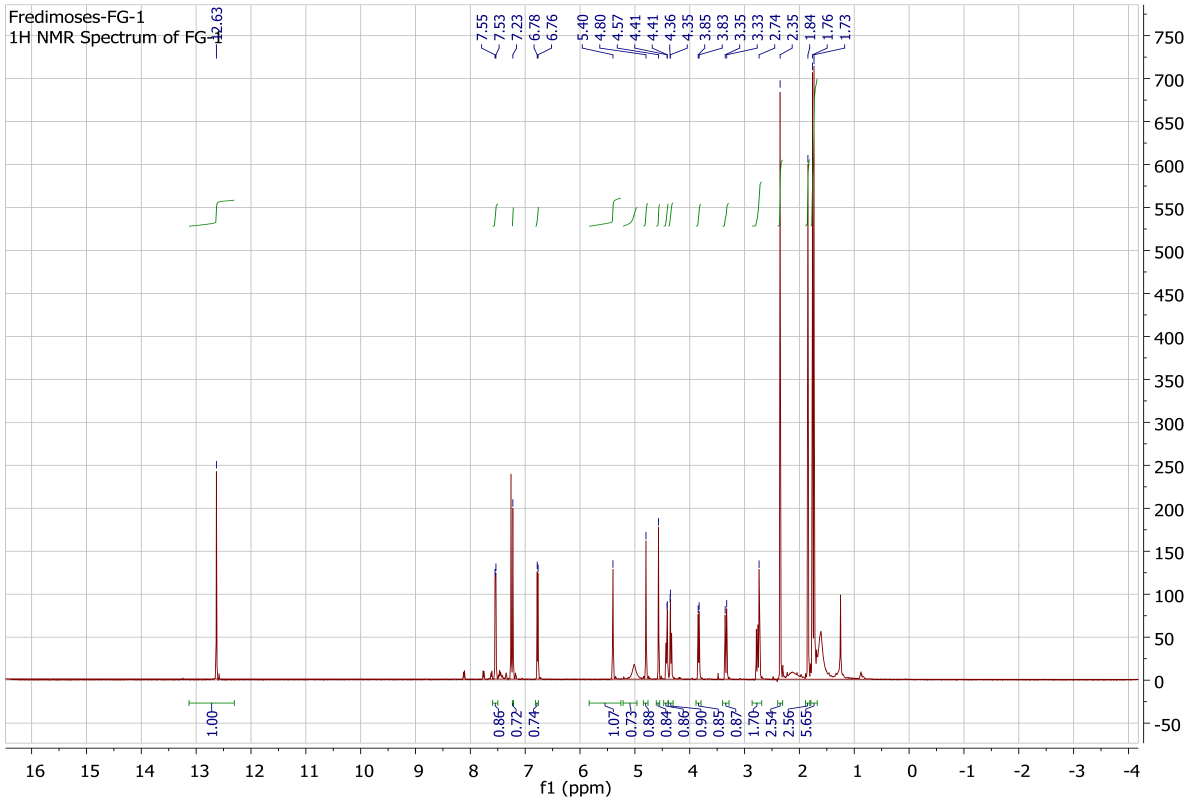

Figure S2. ^{13}C NMR (125 MHz, $CDCl_3$) spectrum of compound **1**.

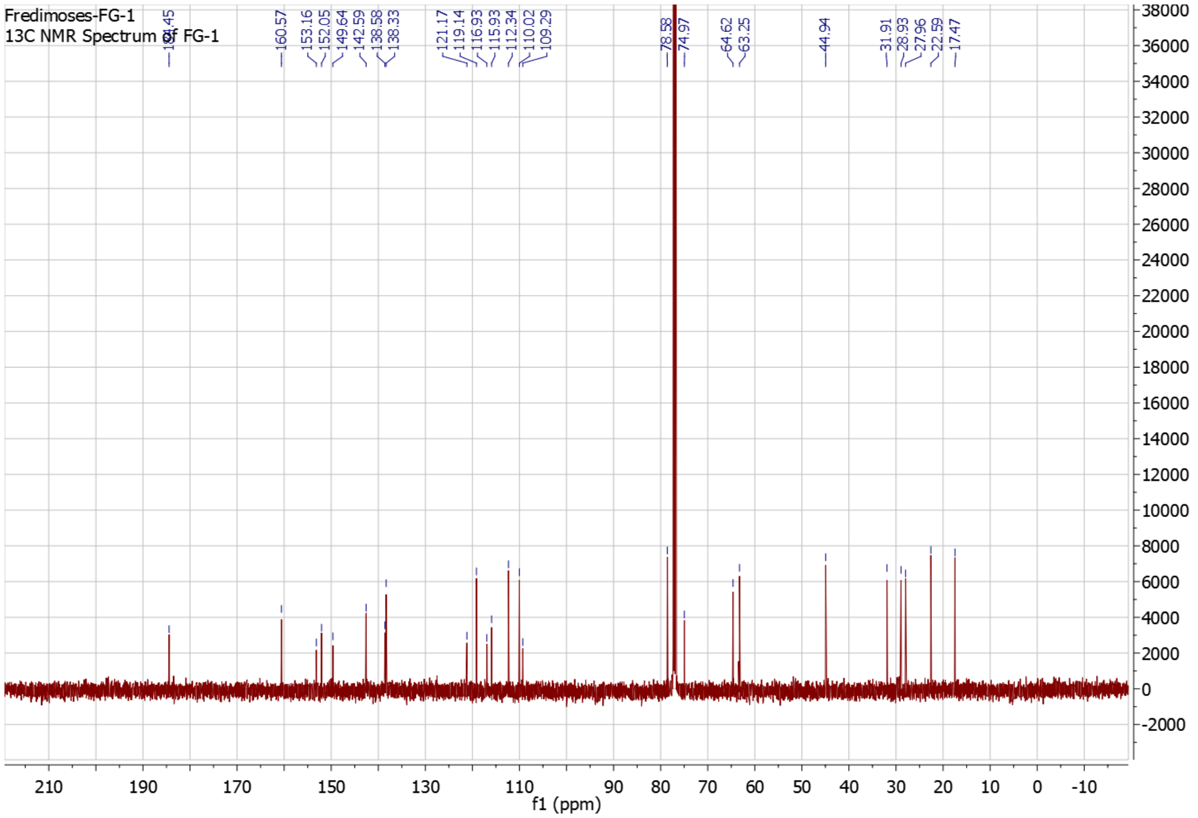

Figure S3. Dept 135 (125 MHz, $CDCl_3$) spectrum of compound **1**.

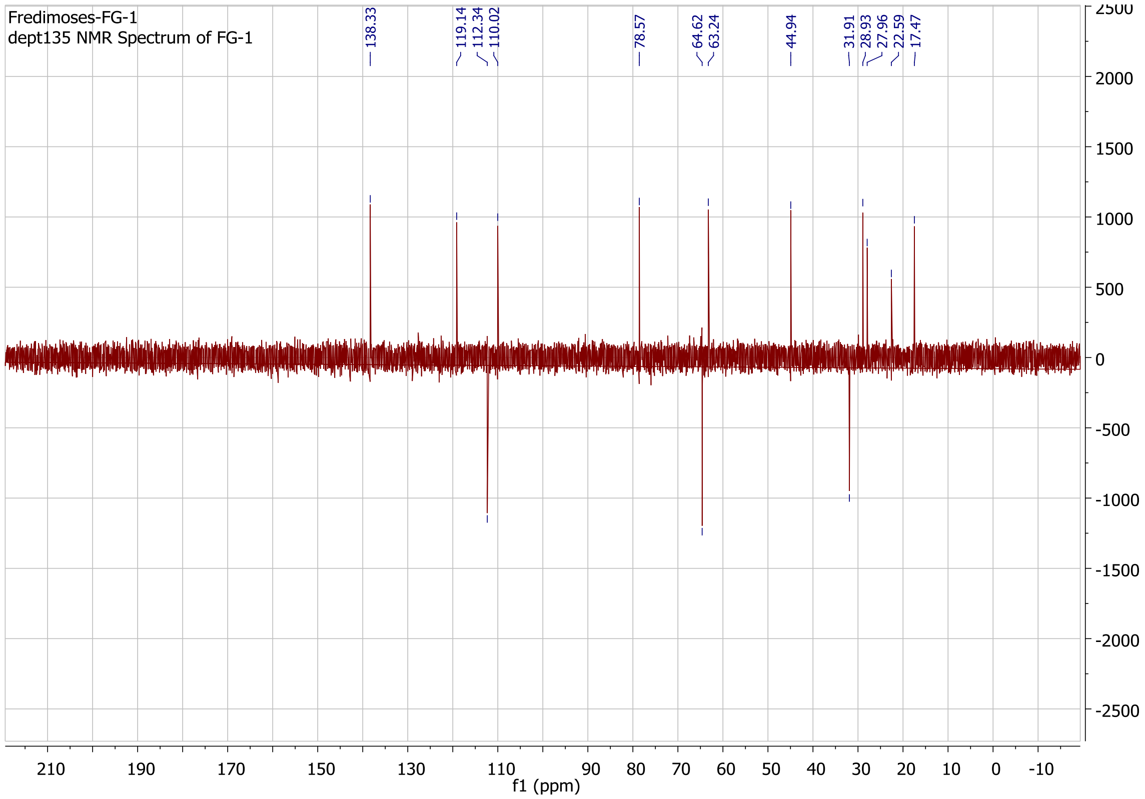

Figure S4. HSQC spectrum of compound **1**.

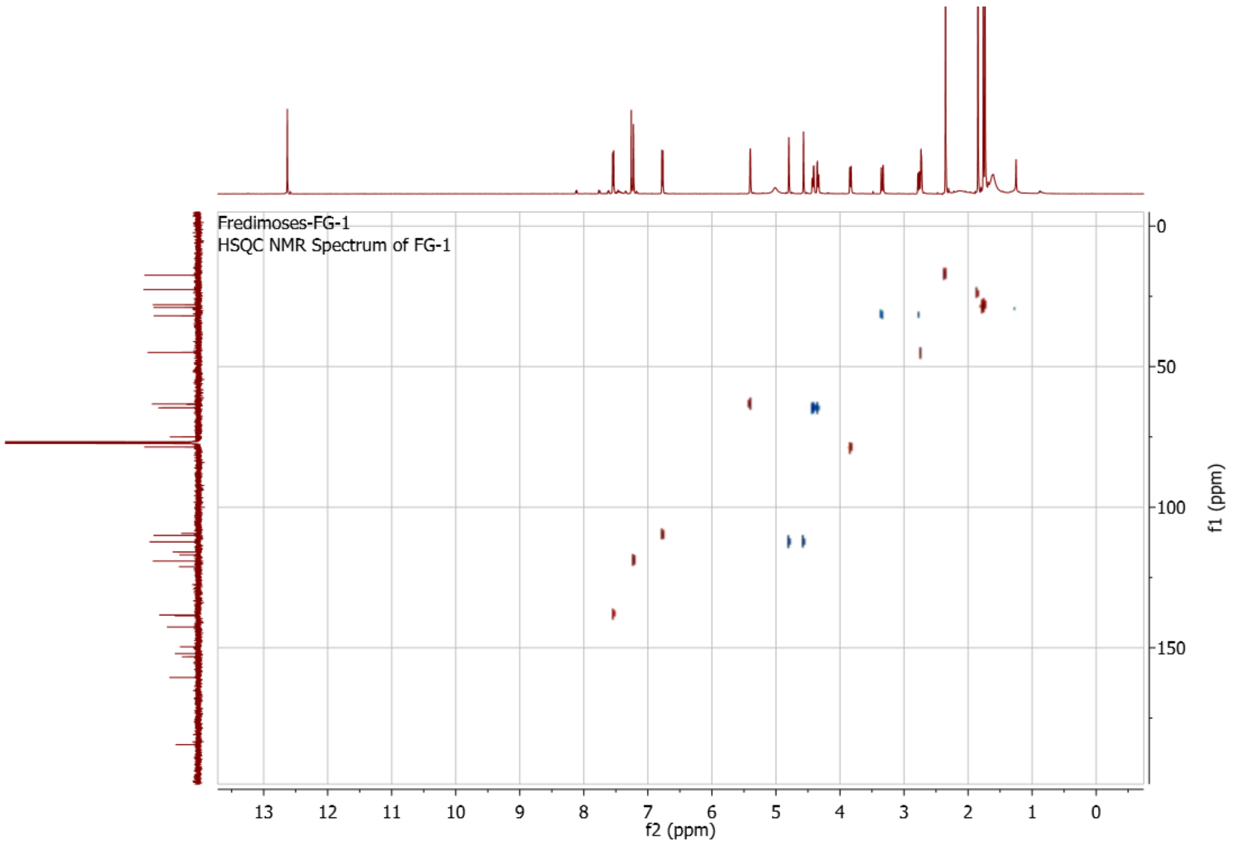

Figure S5. HMBC spectrum of compound **1**.

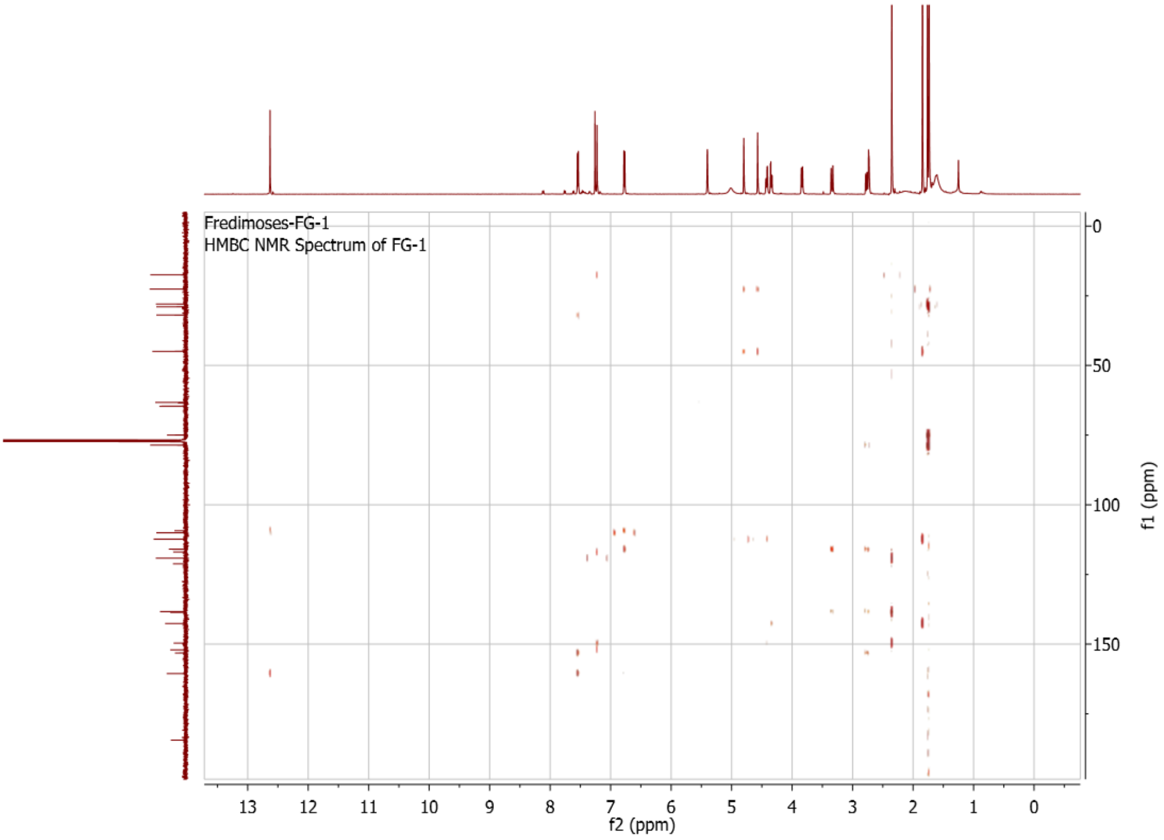

Figure S6. ^{1}H-^{1}H COSY spectrum of compound **1**.

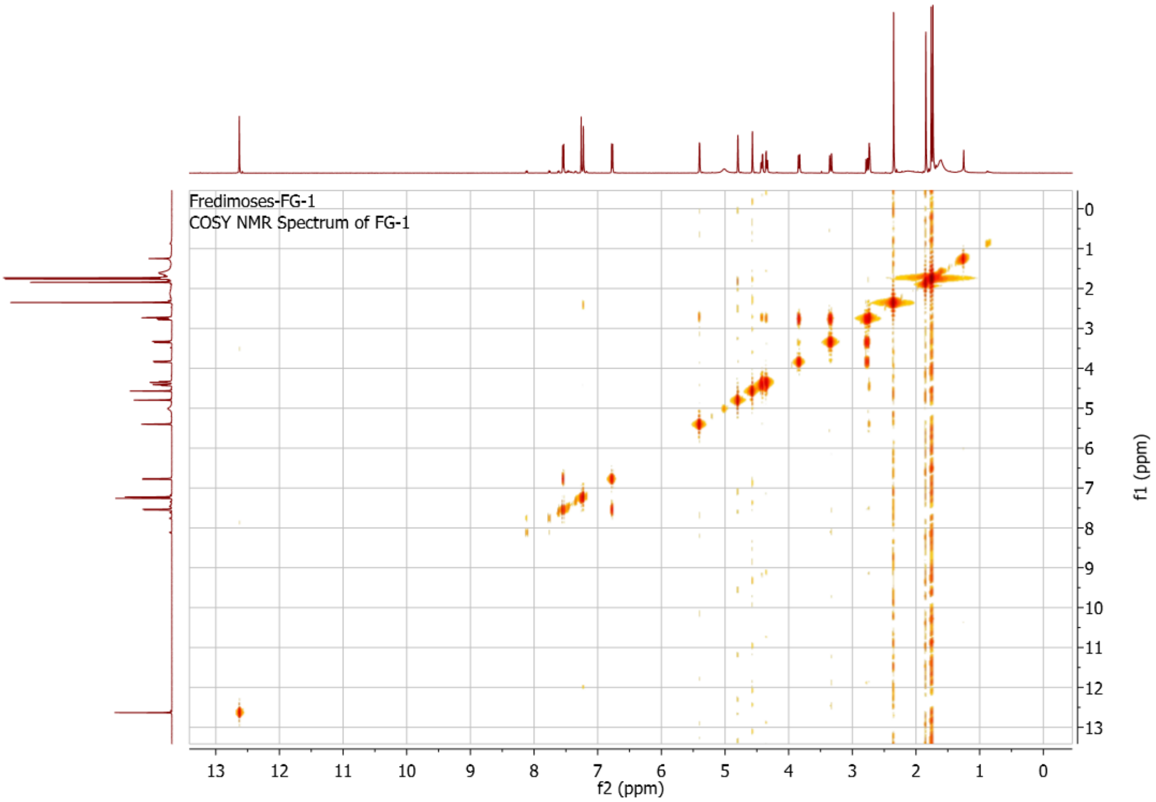

Figure S7. UV spectrum of compound **1**.

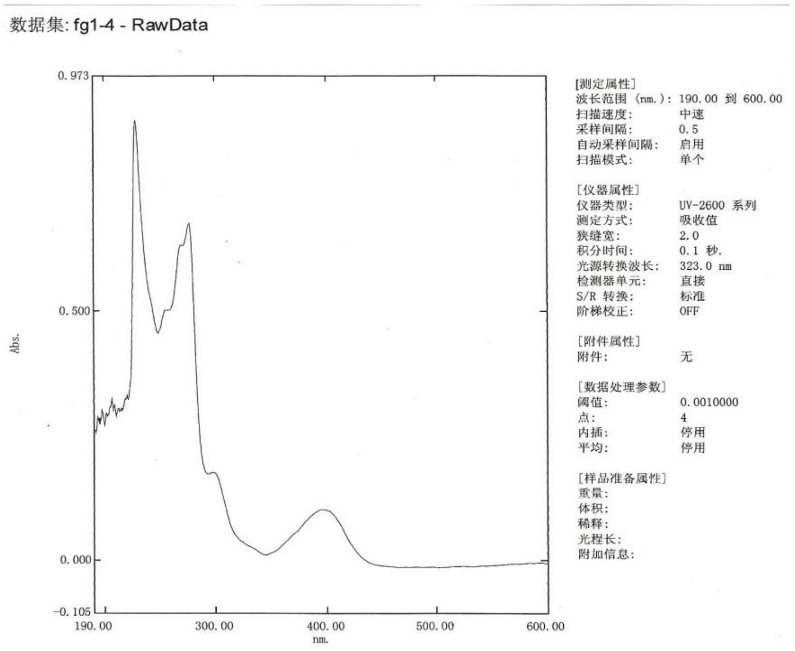

No.	P/V	波长(nm)	吸收值	描述
1	↑	592.50	−0.004	
2	↑	542.00	−0.011	
3	↑	395.50	0.102	
4	↑	298.00	0.176	
5	↑	277.00	0.677	
6	↑	228.50	0.883	
7	↓	532.00	−0.014	
8	↓	464.00	−0.014	
9	↓	345.00	0.011	
10	↓	294.00	0.173	
11	↓	248.50	0.457	
12	↓	200.00	0.270	

Figure S8. IR spectrum of compound **1**.

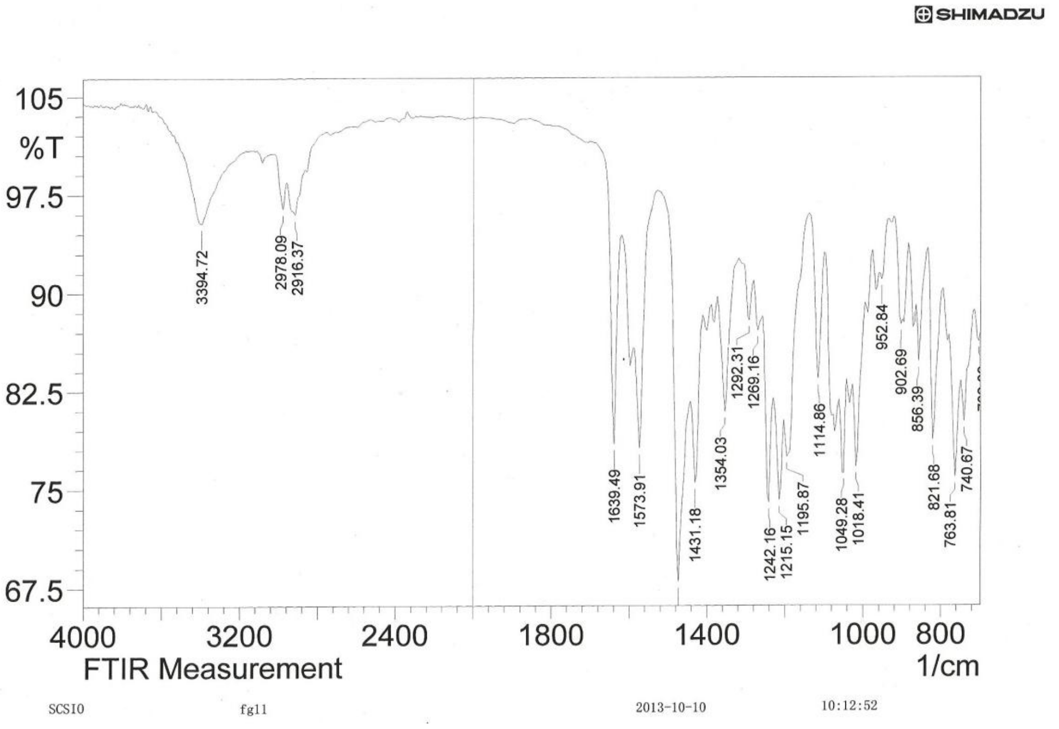

Figure S9. HRESI-MS of compound **1**.

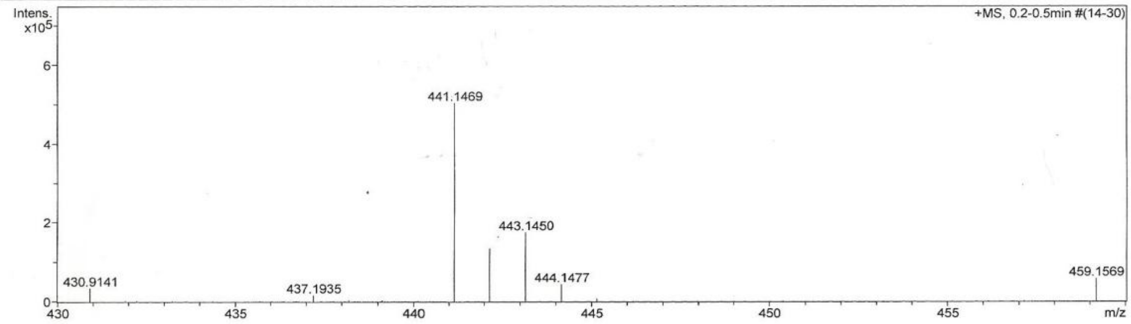

Mass Spectrum SmartFormula Report

Analysis Info

Analysis Name	D:\Data\MS\data\201306\Fredimoses-FG-1_pos.d	Acquisition Date	6/21/2013 9:57:04 AM
Method	POS_100-2000_Dirrect Infusion.m	Operator	SCSIO
Sample Name	SCSIO	Instrument / Ser#	maXis 29
Comment			

Acquisition Parameter

Source Type	ESI	Ion Polarity	Positive	Set Nebulizer	0.3 Bar
Focus	Not active	Set Capillary	3800 V	Set Dry Heater	180 °C
Scan Begin	100 m/z	Set End Plate Offset	-500 V	Set Dry Gas	4.0 l/min
Scan End	2000 m/z	Set Collision Cell RF	2000.0 Vpp	Set Divert Valve	Waste

Meas. m/z	#	Formula	Score	m/z	err [mDa]	err [ppm]	mSigma	rdb	e⁻ Conf	N-Rule
441.1469	1	C 25 H 26 Cl O 5	100.00	441.1463	-0.5	-1.2	8.2	12.5	even	ok
	2	C 26 H 22 Cl N 4 O	76.10	441.1477	0.8	1.8	15.5	17.5	even	ok
881.2864	1	C 50 H 51 Cl 2 O 10	86.74	881.2854	-1.0	-1.1	27.3	24.5	even	ok
	2	C 48 H 39 Cl 2 N 14	77.57	881.2854	-1.0	-1.1	31.7	35.5	even	ok
	3	C 38 H 55 Cl 2 N 2 O 17	69.21	881.2872	0.9	1.0	39.1	11.5	even	ok
	4	C 52 H 43 Cl 2 N 8 O 2	34.26	881.2881	1.7	1.9	45.8	34.5	even	ok

Bruker Compass DataAnalysis 4.0 printed: 6/21/2013 10:07:13 AM Page 1 of 1

Figure S10. ^{1}H NMR (500 MHz, $CDCl_3$) spectrum of compound **2**.

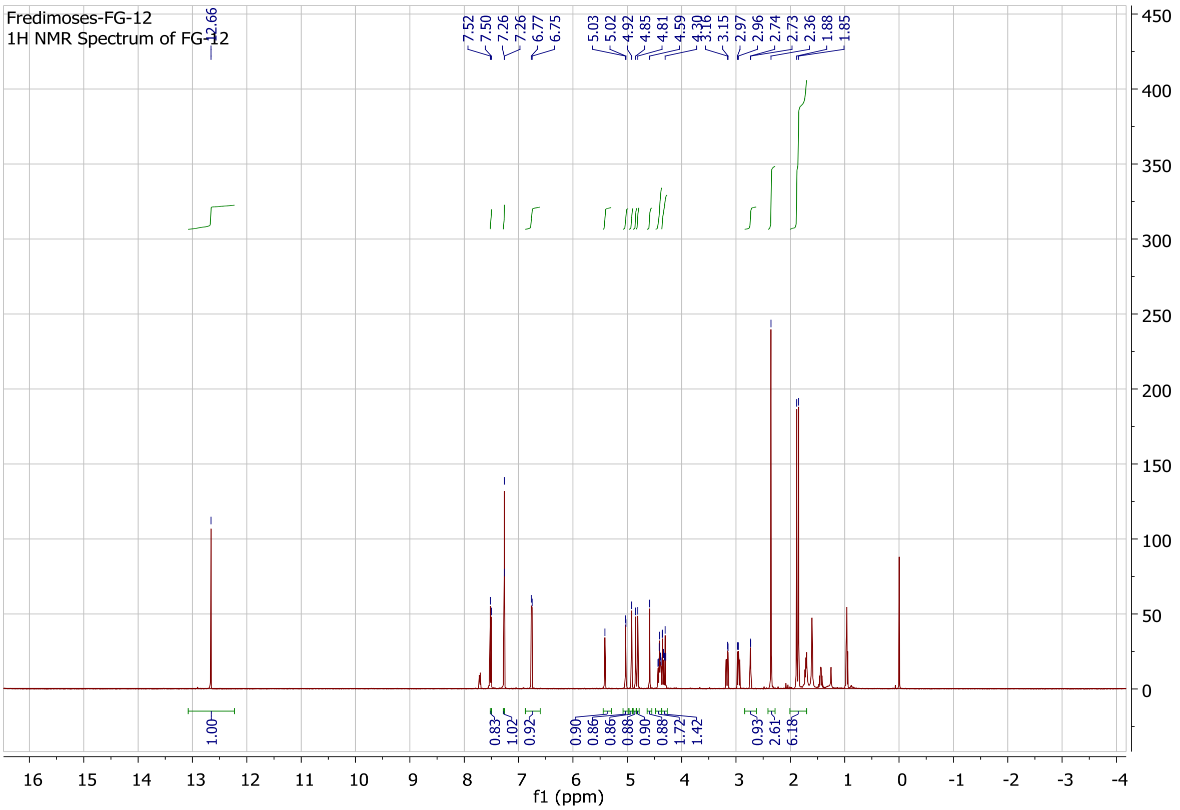

Figure S11. ^{13}C NMR (125 MHz, $CDCl_3$) spectrum of compound **2**.

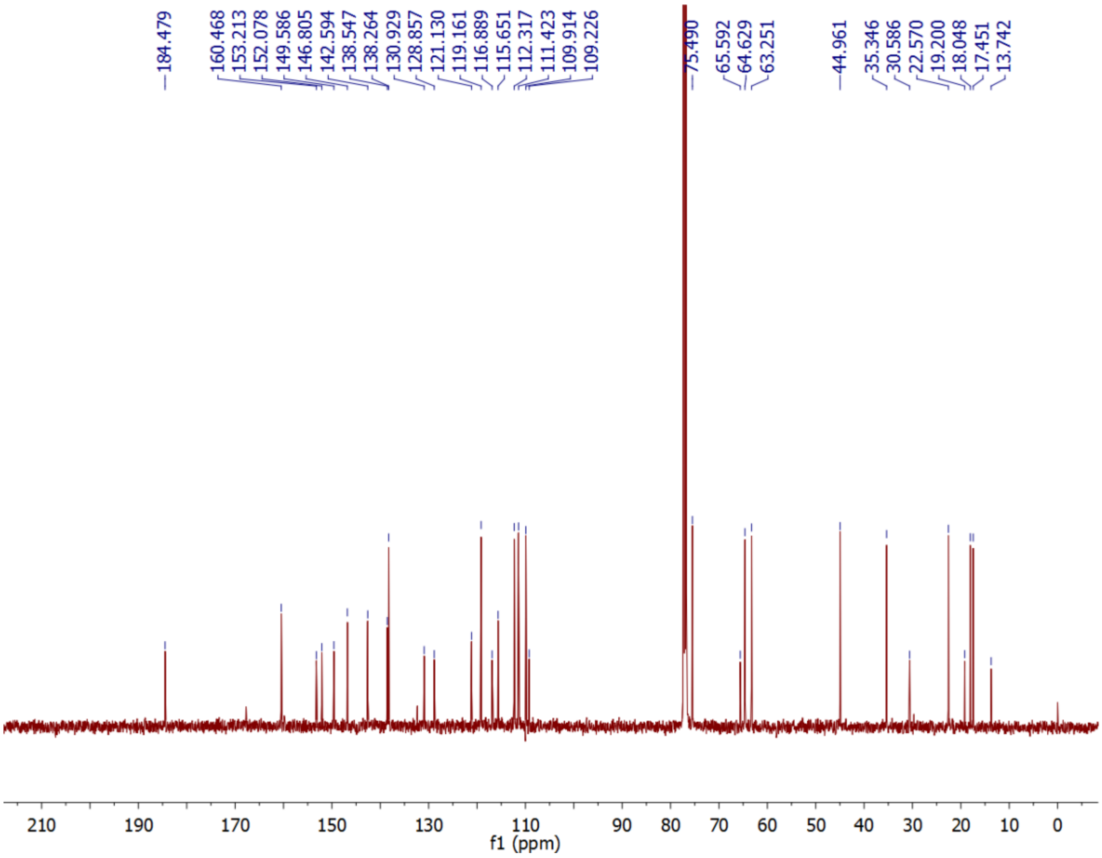

Figure S12. Dept 135 (125 MHz, $CDCl_3$) spectrum of compound **2**.

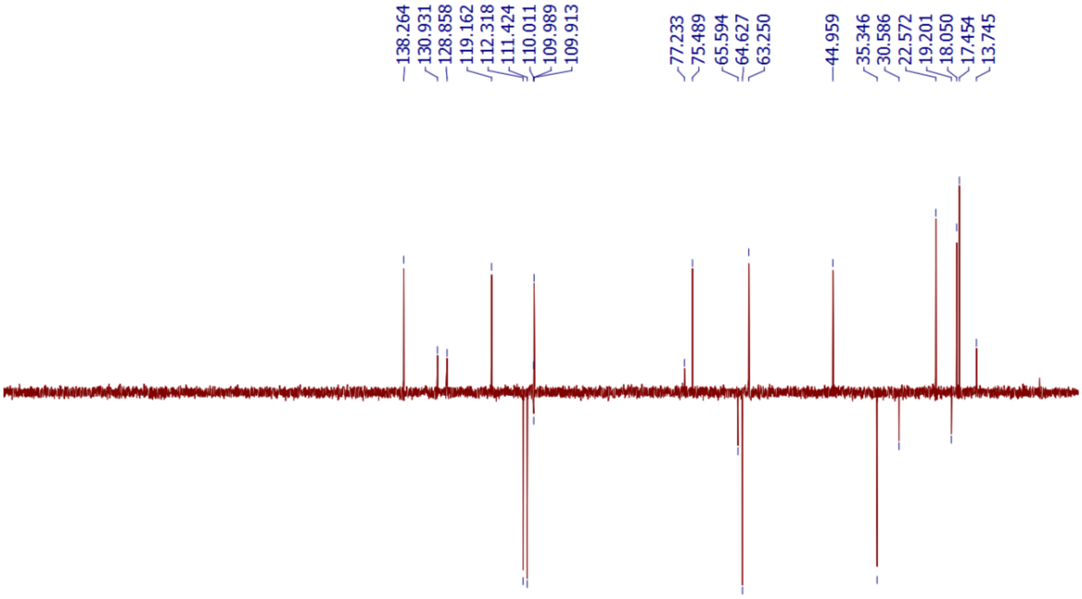

Figure S13. UV spectrum of compound **2**.

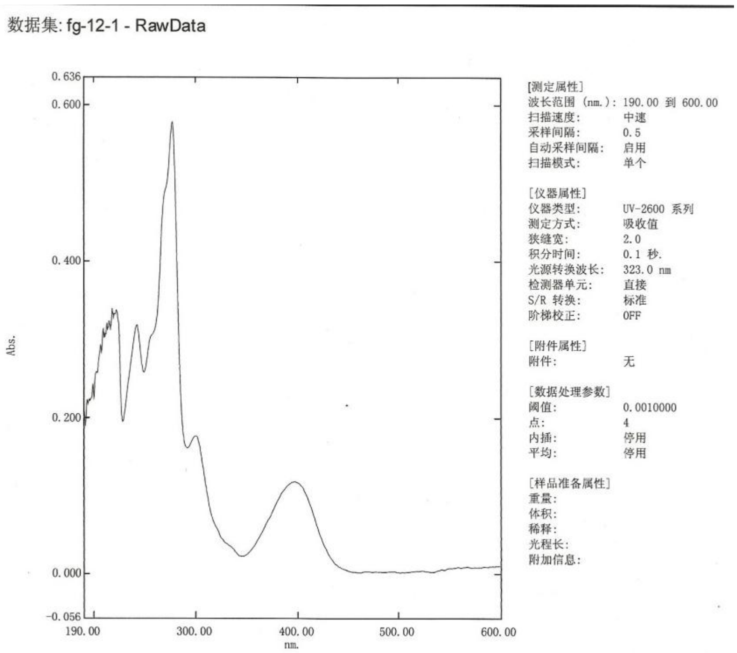

No.	P/V	波长(nm)	吸收值	描述
1	↑	558.50	0.009	
2	↑	524.00	0.004	
3	↑	468.50	0.003	
4	↑	397.00	0.118	
5	↑	300.00	0.177	
6	↑	277.50	0.578	
7	↑	242.50	0.318	
8	↑	218.00	0.338	
9	↓	572.00	0.008	
10	↓	561.50	0.008	
11	↓	532.50	0.002	
12	↓	462.50	0.002	
13	↓	346.00	0.023	
14	↓	292.00	0.161	
15	↓	249.50	0.258	
16	↓	228.50	0.195	

Figure S14. IR spectrum of compound **2**.

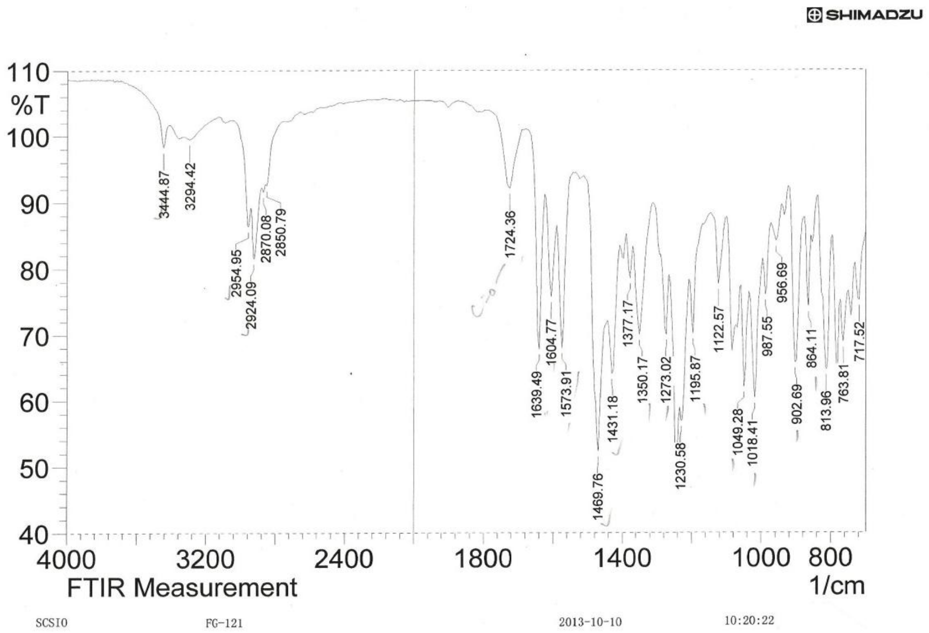

Figure S15. HRESI-MS of compound **2**.

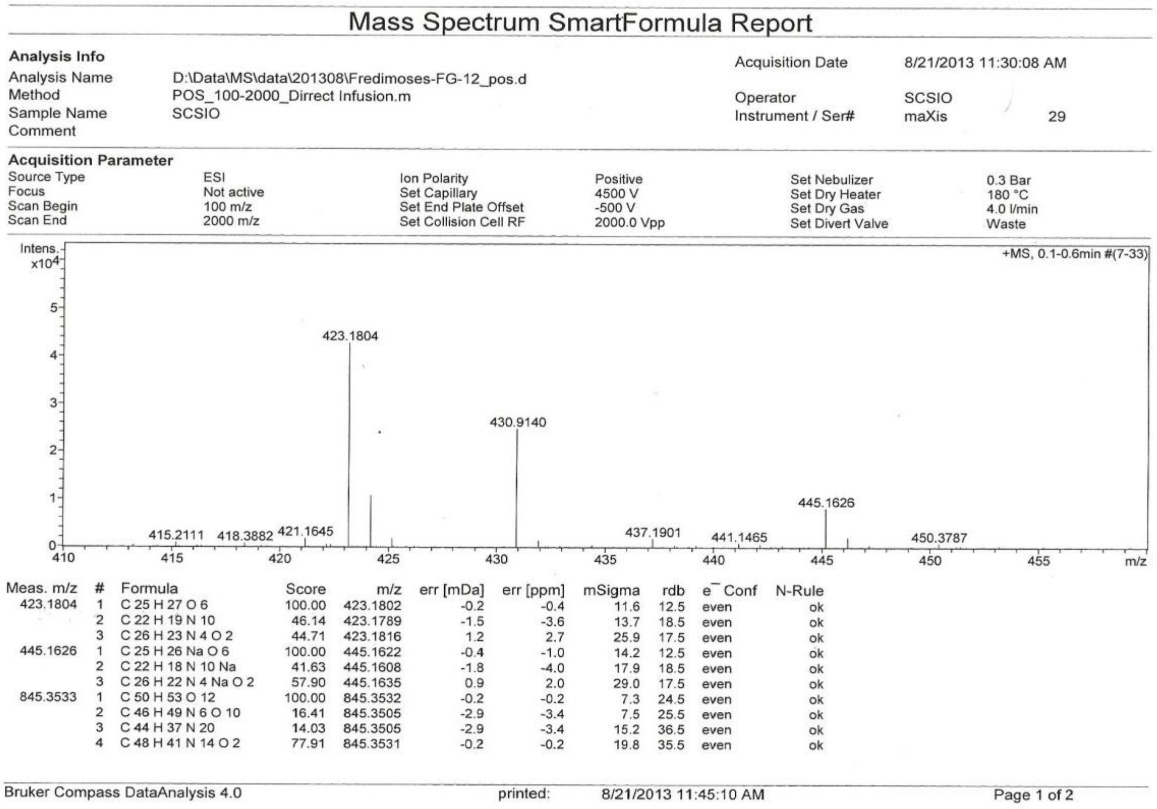

Mass Spectrum SmartFormula Report

Analysis Info

Analysis Name	D:\Data\MS\data\201308\Fredimoses-FG-12_pos.d	Acquisition Date	8/21/2013 11:30:08 AM
Method	POS_100-2000_Dirrect Infusion.m	Operator	SCSIO
Sample Name	SCSIO	Instrument / Ser#	maXis 29
Comment			

Acquisition Parameter

Source Type	ESI	Ion Polarity	Positive	Set Nebulizer	0.3 Bar
Focus	Not active	Set Capillary	4500 V	Set Dry Heater	180 °C
Scan Begin	100 m/z	Set End Plate Offset	-500 V	Set Dry Gas	4.0 l/min
Scan End	2000 m/z	Set Collision Cell RF	2000.0 Vpp	Set Divert Valve	Waste

Meas. m/z	#	Formula	Score	m/z	err [mDa]	err [ppm]	mSigma	rdb	e⁻ Conf	N-Rule
423.1804	1	C 25 H 27 O 6	100.00	423.1802	-0.2	-0.4	11.6	12.5	even	ok
	2	C 22 H 19 N 10	46.14	423.1789	-1.5	-3.6	13.7	18.5	even	ok
	3	C 26 H 23 N 4 O 2	44.71	423.1816	1.2	2.7	25.9	17.5	even	ok
445.1626	1	C 25 H 26 Na O 6	100.00	445.1622	-0.4	-1.0	14.2	12.5	even	ok
	2	C 22 H 18 N 10 Na	41.63	445.1608	-1.8	-4.0	17.9	18.5	even	ok
	3	C 26 H 22 N 4 Na O 2	57.90	445.1635	0.9	2.0	29.0	17.5	even	ok
845.3533	1	C 50 H 53 O 12	100.00	845.3532	-0.2	-0.2	7.3	24.5	even	ok
	2	C 46 H 49 N 6 O 10	16.41	845.3505	-2.9	-3.4	7.5	25.5	even	ok
	3	C 44 H 37 N 20	14.03	845.3505	-2.9	-3.4	15.2	36.5	even	ok
	4	C 48 H 41 N 14 O 2	77.91	845.3531	-0.2	-0.2	19.8	35.5	even	ok

Bruker Compass DataAnalysis 4.0 printed: 8/21/2013 11:45:10 AM Page 1 of 2

Figure S16. ^{1}H NMR (500 MHz, $CDCl_3$) spectrum of compound **3**.

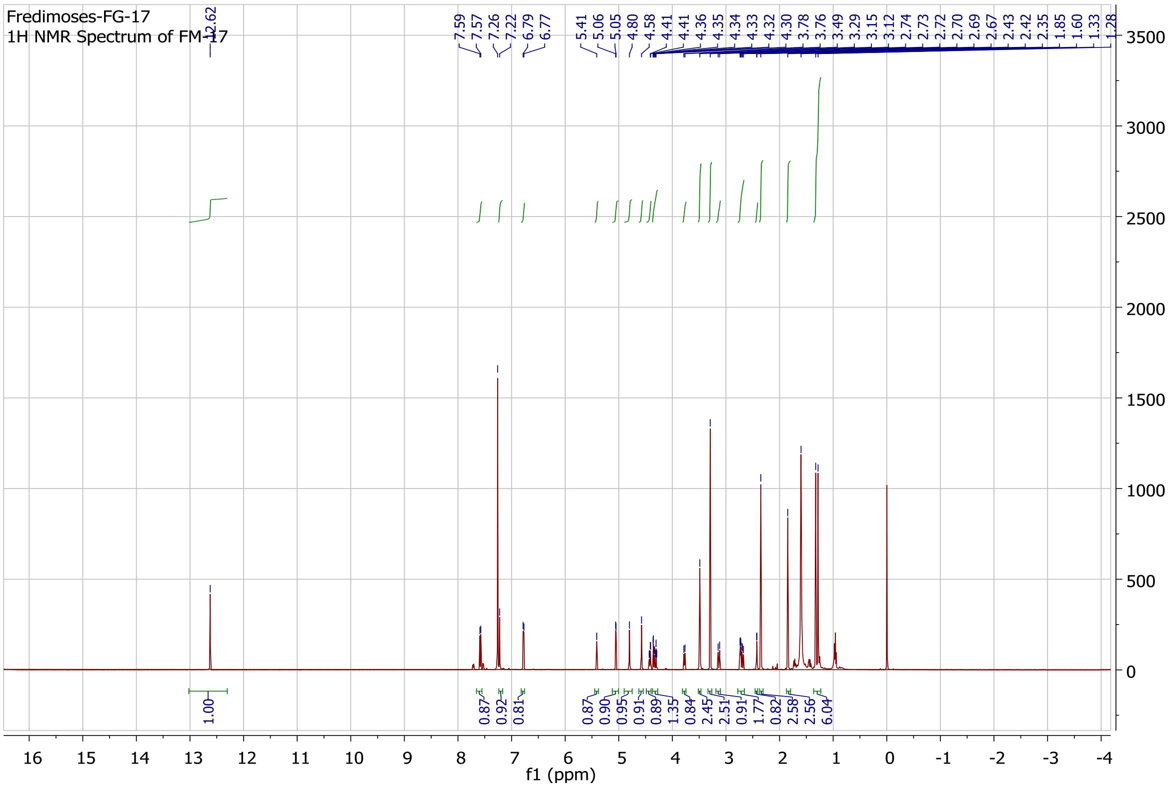

Figure S17. ^{13}C NMR (125 MHz, $CDCl_3$) spectrum of compound **3**.

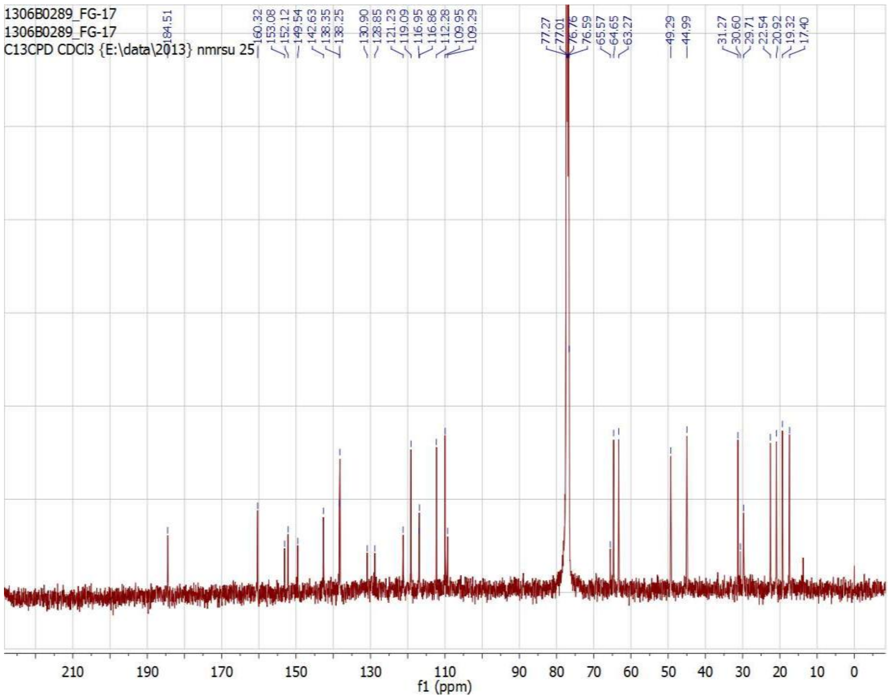

Figure S18. UV spectrum of compound **3**.

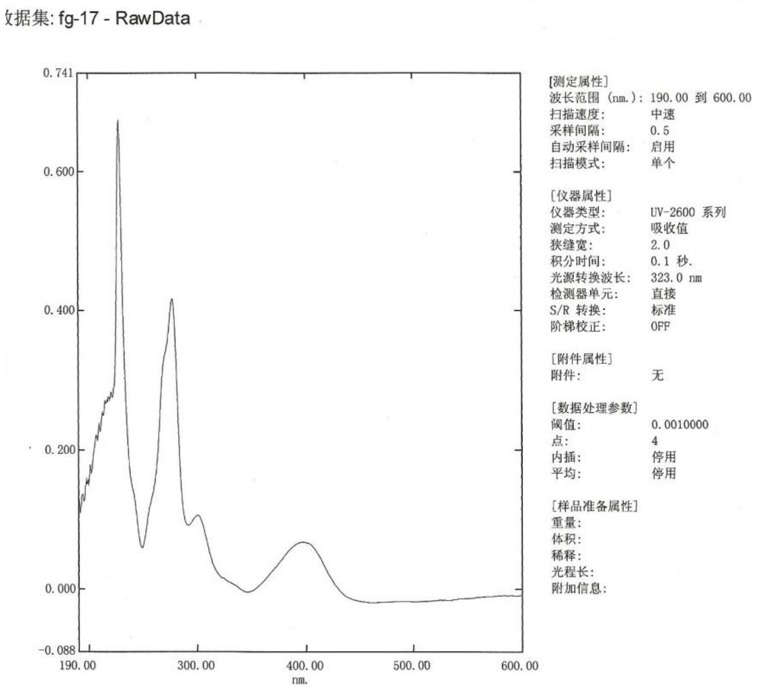

No.	P/V	波长(nm)	吸收值	描述
1	↑	398.50	0.068	
2	↑	300.50	0.106	
3	↑	277.50	0.417	
4	↑	228.00	0.672	
5	↓	532.00	−0.016	
6	↓	502.00	−0.018	
7	↓	463.00	−0.019	
8	↓	346.50	−0.004	
9	↓	291.50	0.092	
10	↓	249.00	0.060	

Figure S19. IR spectrum of compound **3**.

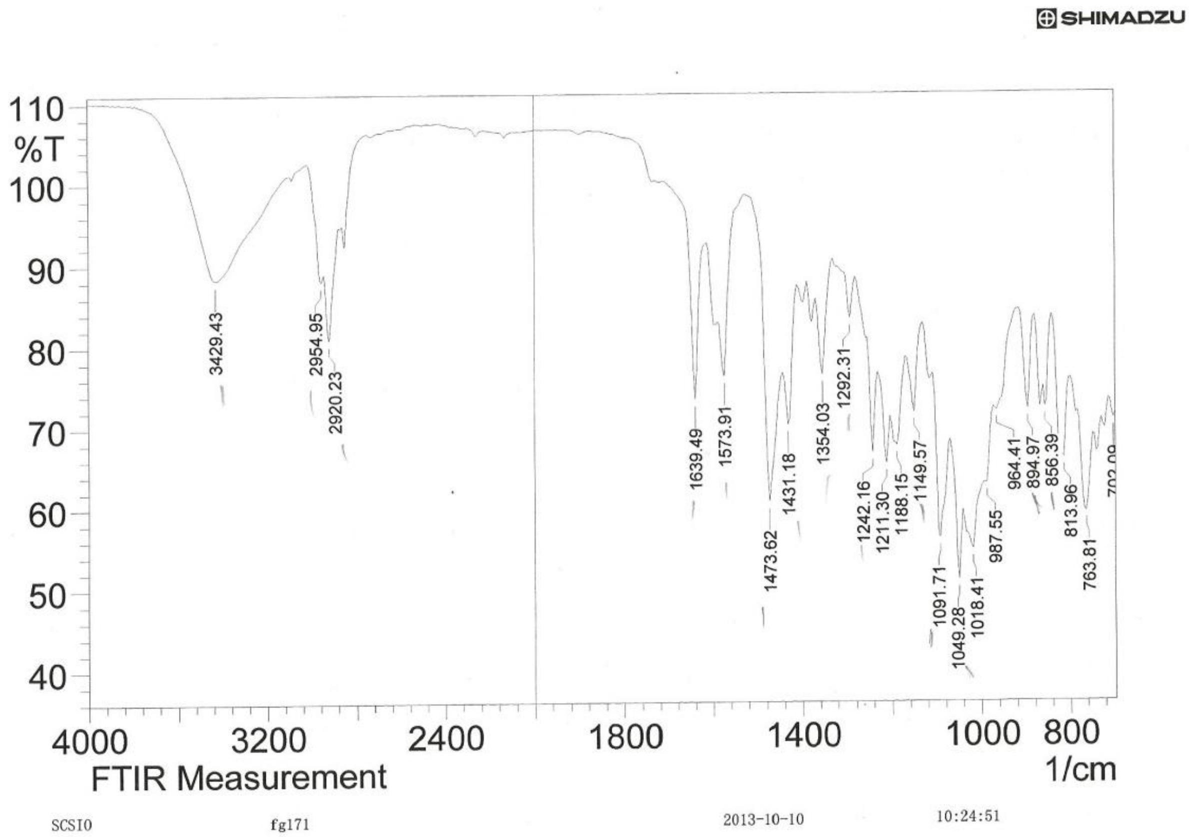

Figure S20. HRESI-MS of compound **3**.

Mass Spectrum SmartFormula Report

Analysis Info

Analysis Name	D:\Data\MS\data\201306\Fredimoses_FG-17_pos.d	Acquisition Date	6/26/2013 3:48:21 PM
Method	POS_100-2000_Dirrect Infusion.m	Operator	SCSIO
Sample Name	SCSIO	Instrument / Ser#	maXis 29
Comment			

Acquisition Parameter

Source Type	ESI	Ion Polarity	Positive	Set Nebulizer	0.3 Bar
Focus	Not active	Set Capillary	3800 V	Set Dry Heater	180 °C
Scan Begin	100 m/z	Set End Plate Offset	-500 V	Set Dry Gas	2.0 l/min
Scan End	2000 m/z	Set Collision Cell RF	2000.0 Vpp	Set Divert Valve	Waste

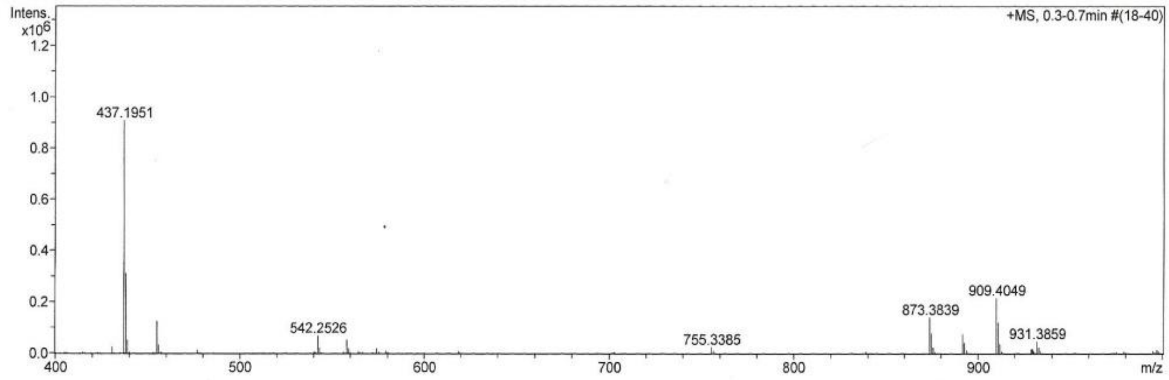

Meas. m/z	#	Formula	Score	m/z	err [mDa]	err [ppm]	mSigma	rdb	e⁻ Conf	N-Rule
455.2055	1	C 26 H 31 O 7	100.00	455.2064	0.9	2.0	15.8	11.5	even	ok
477.1870	1	C 26 H 30 Na O 7	100.00	477.1884	1.3	2.8	11.3	11.5	even	ok
909.4049	1	C 52 H 61 O 14	100.00	909.4056	0.7	0.8	12.9	22.5	even	ok
931.3859	1	C 52 H 60 Na O 14	100.00	931.3875	1.6	1.7	23.8	22.5	even	ok

Figure S21. ^{1}H NMR (500 MHz, $CDCl_3$) spectrum of compound **4**.

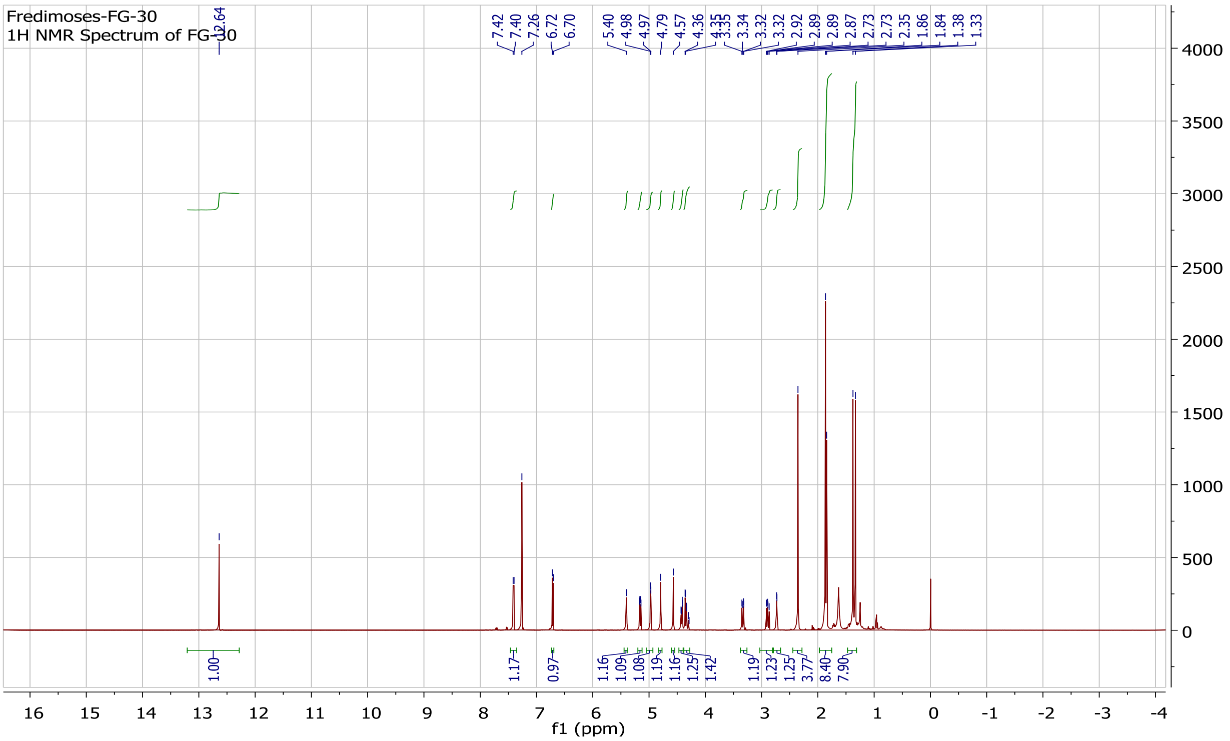

Figure S22. ^{13}C NMR (125 MHz, $CDCl_3$) spectrum of compound **4**.

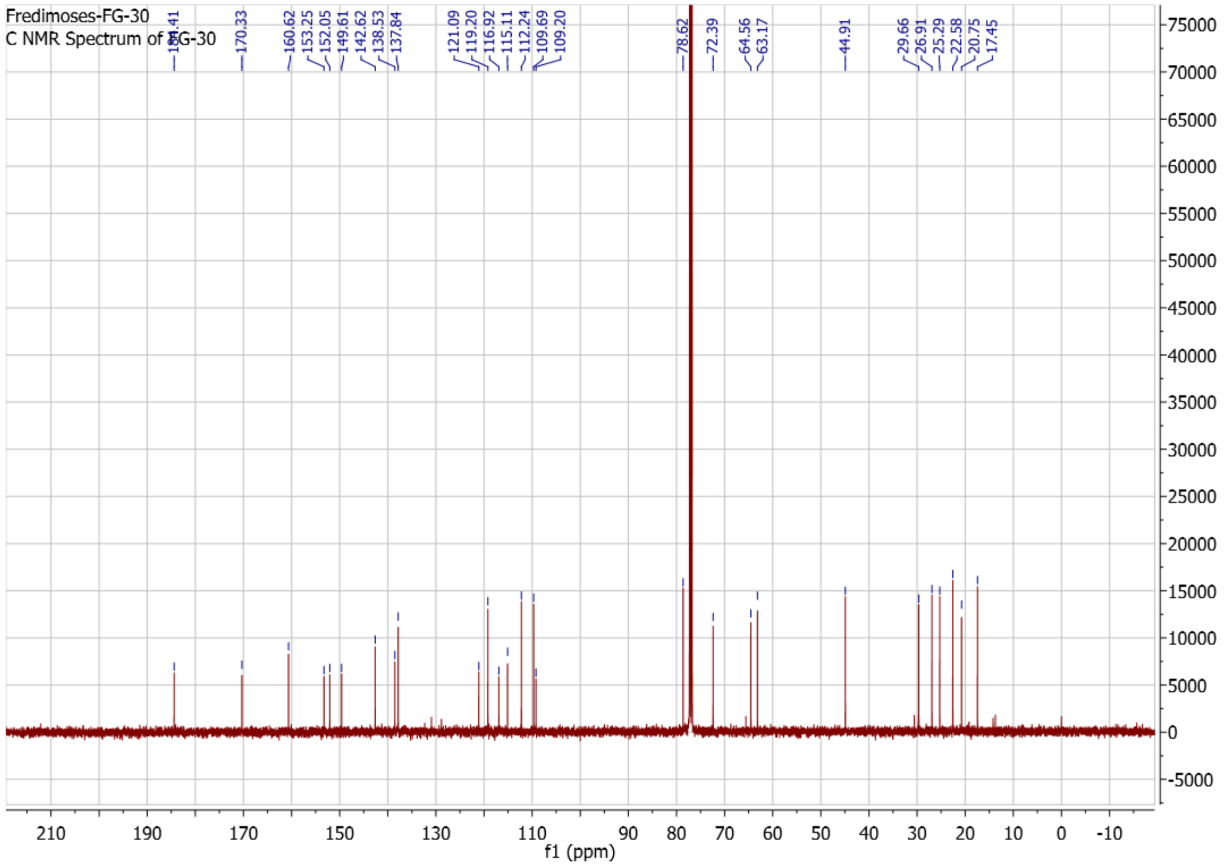

Figure S23. Dept 135 (125 MHz, $CDCl_3$) spectrum of compound **4**.

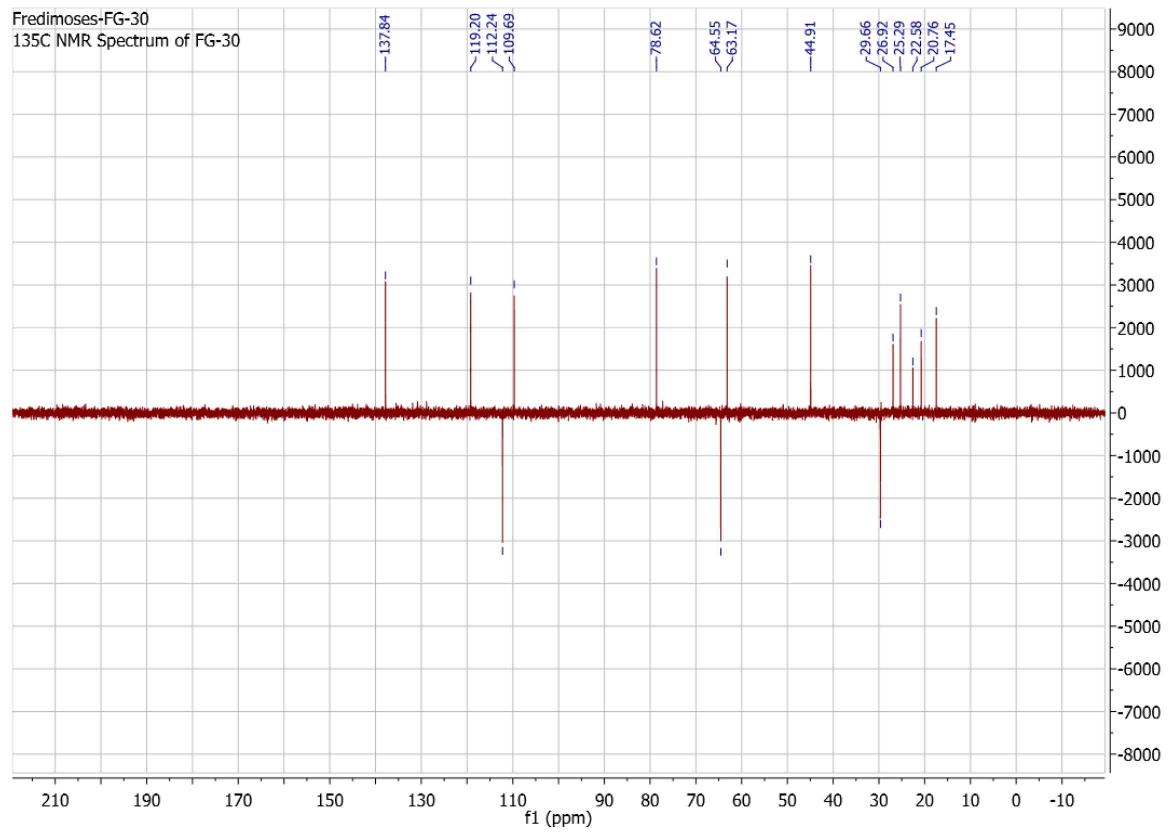

Figure S24. HSQC spectrum of compound **4**.

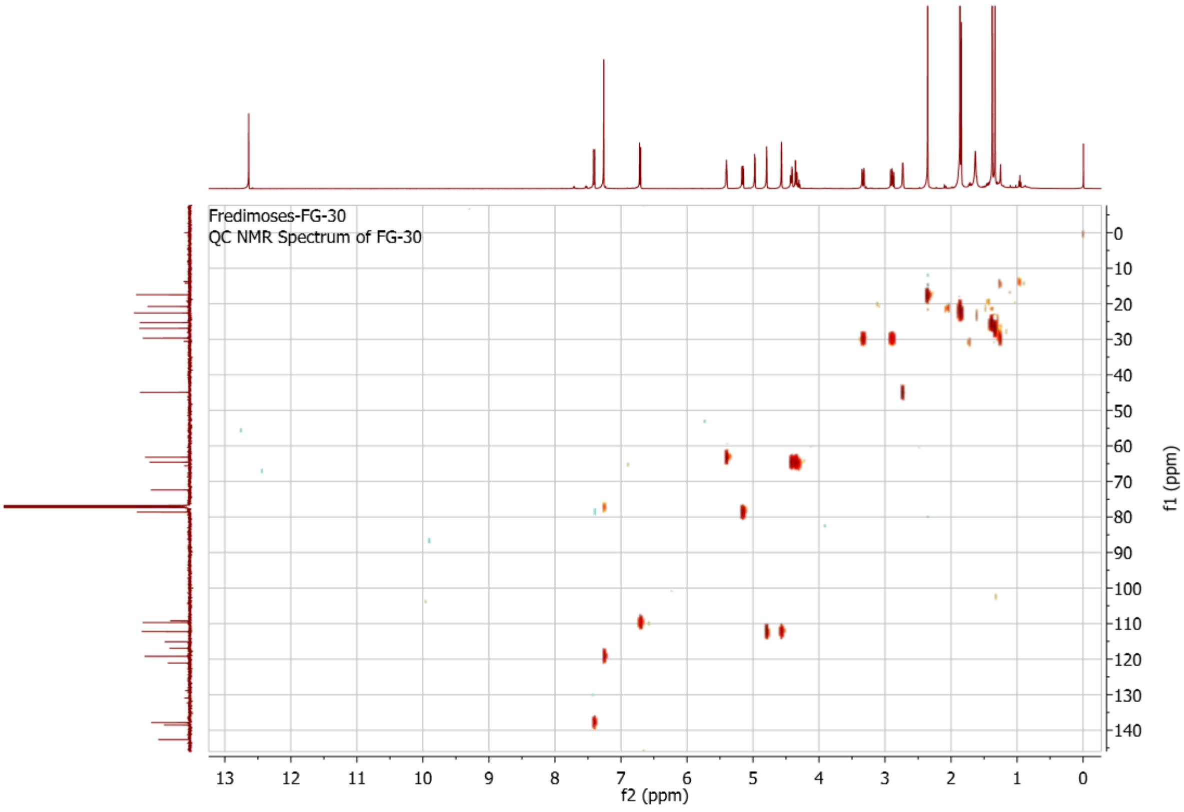

Figure S25. HMBC spectrum of compound **4**.

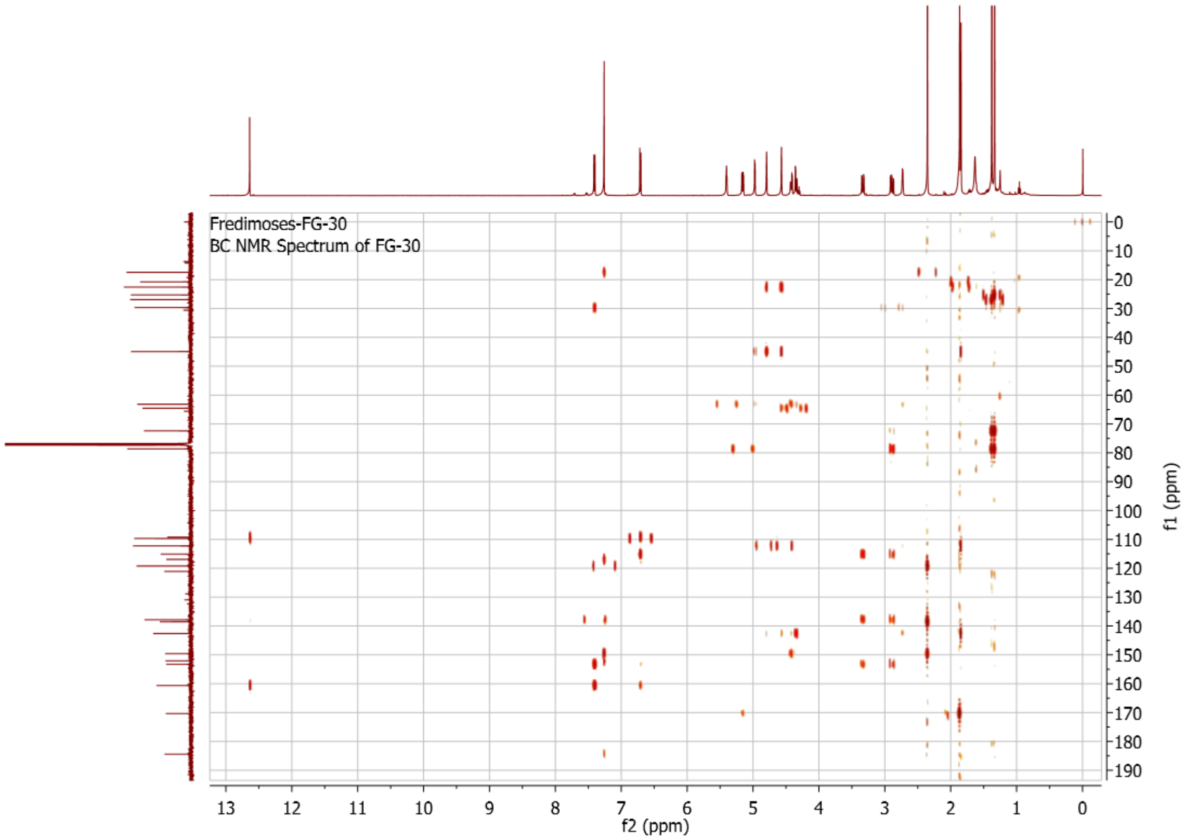

Figure S26. UV spectrum of compound **4**.

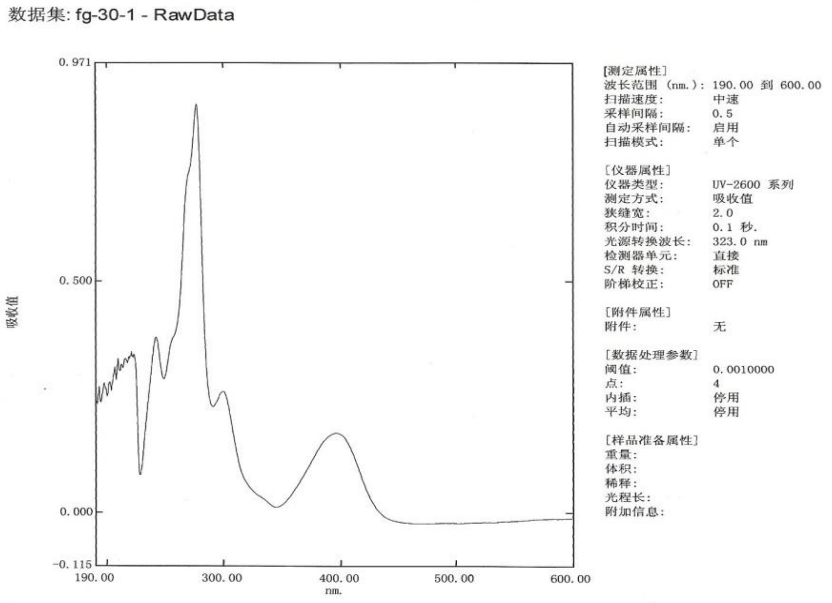

No.	P/V	波长(nm)	吸收值	描述
1	↑	396. 50	0. 171	
2	↑	299. 50	0. 260	
3	↑	277. 00	0. 880	
4	↑	242. 50	0. 377	
5	↑	221. 00	0. 345	
6	↑	197. 50	0. 278	
7	↓	345. 00	0. 011	
8	↓	291. 00	0. 227	
9	↓	249. 00	0. 287	
10	↓	228. 00	0. 081	
11	↓	200. 00	0. 250	

Figure S27. IR spectrum of compound **4**.

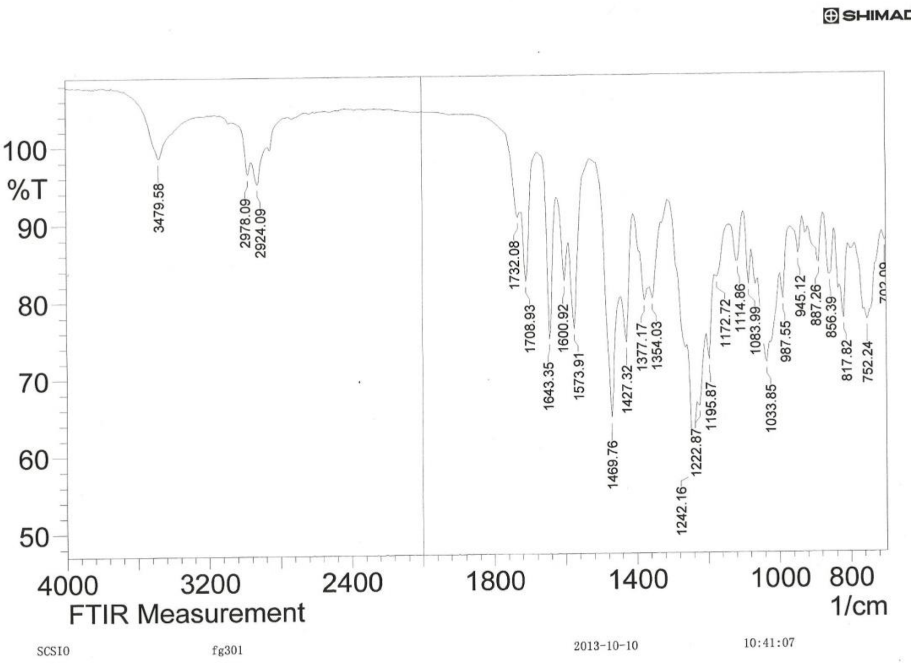

Figure S28. HRESI-MS of compound **4**.

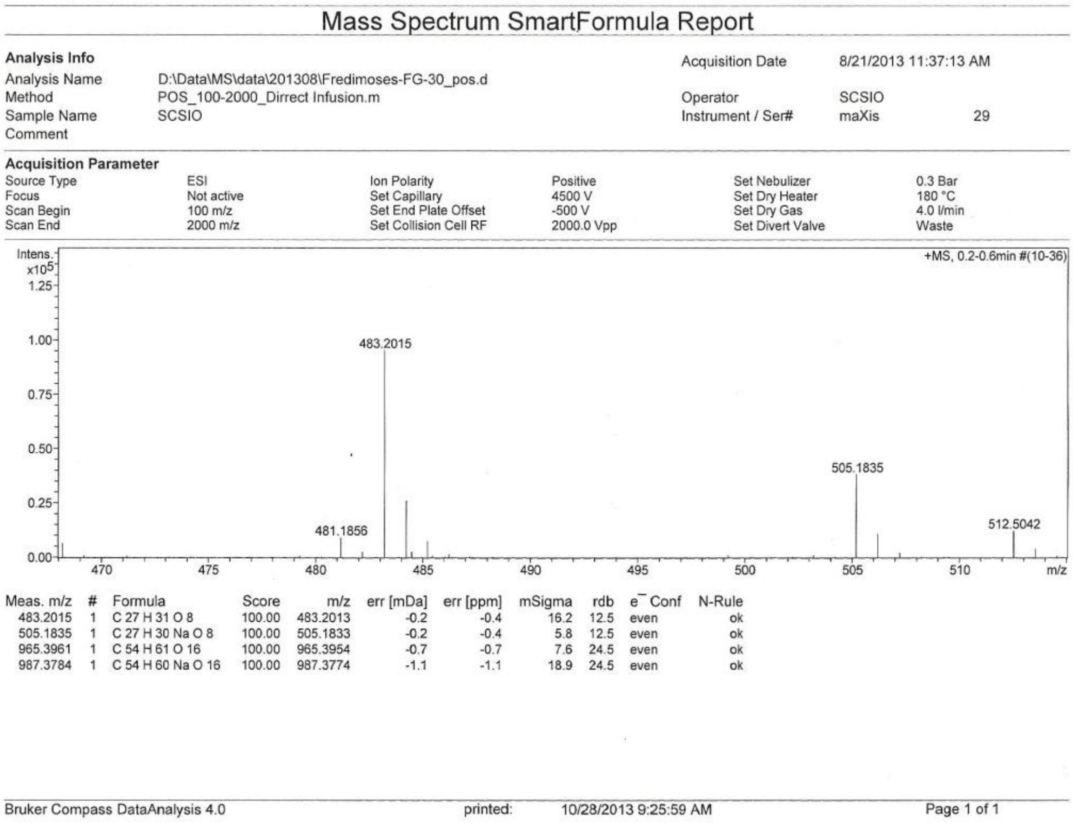

Mass Spectrum SmartFormula Report

Analysis Info

Analysis Name	D:\Data\MS\data\201308\Fredimoses-FG-30_pos.d	Acquisition Date	8/21/2013 11:37:13 AM
Method	POS_100-2000_Dirrect Infusion.m	Operator	SCSIO
Sample Name	SCSIO	Instrument / Ser#	maXis 29
Comment			

Acquisition Parameter

Source Type	ESI	Ion Polarity	Positive	Set Nebulizer	0.3 Bar
Focus	Not active	Set Capillary	4500 V	Set Dry Heater	180 °C
Scan Begin	100 m/z	Set End Plate Offset	-500 V	Set Dry Gas	4.0 l/min
Scan End	2000 m/z	Set Collision Cell RF	2000.0 Vpp	Set Divert Valve	Waste

Meas. m/z	#	Formula	Score	m/z	err [mDa]	err [ppm]	mSigma	rdb	e⁻ Conf	N-Rule
483.2015	1	C 27 H 31 O 8	100.00	483.2013	-0.2	-0.4	16.2	12.5	even	ok
505.1835	1	C 27 H 30 Na O 8	100.00	505.1833	-0.2	-0.4	5.8	12.5	even	ok
965.3961	1	C 54 H 61 O 16	100.00	965.3954	-0.7	-0.7	7.6	24.5	even	ok
987.3784	1	C 54 H 60 Na O 16	100.00	987.3774	-1.1	-1.1	18.9	24.5	even	ok

Bruker Compass DataAnalysis 4.0 printed: 10/28/2013 9:25:59 AM Page 1 of 1

© 2014 by the authors; licensee MDPI, Basel, Switzerland. This article is an open access article distributed under the terms and conditions of the Creative Commons Attribution license (http://creativecommons.org/licenses/by/3.0/).
